# The protein carboxymethyltransferase–dependent aspartate salvage pathway plays a crucial role in the intricate metabolic network of *Escherichia coli*

**DOI:** 10.1126/sciadv.adj0767

**Published:** 2024-02-09

**Authors:** Maureen Micaletto, Sebastien Fleurier, Sara Dion, Erick Denamur, Ivan Matic

**Affiliations:** ^1^ Institut Cochin, Université Paris Cité, INSERM U1016, CNRS UMR 8104, 75014 Paris, France.; ^2^ IAME, Université de Paris, INSERM U1137, Université Sorbonne Paris Nord, 75018 Paris, France.; ^3^ AP-HP, Laboratoire de Génétique Moléculaire, Hôpital Bichat, 75018 Paris, France.

## Abstract

Protein carboxymethyltransferase (Pcm) is a highly evolutionarily conserved enzyme that initiates the conversion of abnormal isoaspartate to aspartate residues. While it is commonly believed that Pcm facilitates the repair of damaged proteins, a number of observations suggest that it may have another role in cell functioning. We investigated whether Pcm provides a means for *Escherichia coli* to recycle aspartate, which is essential for protein synthesis and other cellular processes. We showed that Pcm is required for the energy production, the maintenance of cellular redox potential and of *S*-adenosylmethionine synthesis, which are critical for the proper functioning of many metabolic pathways. Pcm contributes to the full growth capacity both under aerobic and anaerobic conditions. Last, we showed that Pcm enhances the robustness of bacteria when exposed to sublethal antibiotic treatments and improves their fitness in the mammalian urinary tract. We propose that Pcm plays a crucial role in *E. coli* metabolism by ensuring a steady supply of aspartate.

## Teaser

Enzymes, such as Pcm, which repair damaged macromolecules other than DNA, are rare but highly important for cellular functioning.

## INTRODUCTION

Amino acids are intrinsically chemically reactive molecules, which makes them prone to both spontaneous and induced nonenzymatic modifications that can result in conformational and functional changes of proteins (*1*). While approximately 120 covalent modifications of amino acids have been identified (*2*), only three enzymes are known to be dedicated to repairing modified amino acids (*3*): peptidyl prolyl cis trans isomerase (*4*), methionine sulfoxide reductase (*5*), and protein l-isoaspartate *O*-methyltransferase (PIMT) also known as protein carboxymethyltransferase (Pcm) (*6*). These three enzymes are highly evolutionarily conserved (*4*, *7*, *8*). This indicates that, while all proteinogenic amino acids are essential, aspartate, methionine, and proline are special and particularly important for the cell functioning.

Aspartate and asparagine are highly susceptible to nonenzymatic dehydration and deamidation, respectively (*9*, *10*). This results in the formation of unstable succinimide intermediates that rapidly spontaneously hydrolyze into isoaspartate (IsoAsp) (*9*). IsoAsp is processed by the Pcm, which transfers a methyl group from a *S*-adenosylmethionine (SAM) to IsoAsp (fig. S1) (*6*). The resulting l-isoaspartate methyl ester converts spontaneously back into succinimide. The spontaneous hydrolysis of succinimide produces IsoAsp and normal aspartate in 3:1 ratio. This cycle is repeated until the majority of IsoAsp is converted into aspartate (*11*). Although the activity of Pcm is not directly adenosine 5′-triphosphate (ATP)–dependent, repairing IsoAsp is energetically costly because the formation of one SAM requires the hydrolysis of three ATPs (fig. S1) (*12*).

The vast majority of articles describes Pcm as a protein repair enzyme that is particularly important under stress conditions (*6*, *12*, *13*). However, it has been reported that Pcm processing of IsoAsp is more efficient on peptides than on proteins (*14*). Regardless of whether IsoAsp was derived from aspartate or asparagine, the Pcm action always results in the production of aspartate. This results in asparagine to aspartate substitution in protein, which can affect protein function (*15*). These observations imply that Pcm may have a different function apart from protein repair, possibly being involved in the aspartate salvage pathway. This hypothesis is plausible, considering the essential role of aspartate in metabolism. Aspartate plays a pivotal role in bacterial metabolism because it is also a precursor to the biosynthesis of pyrimidines, nicotinamide adenine dinucleotide (NAD), the purine bases—ATP and guanosine 5′-triphosphate (GTP)—and several amino acids, namely, threonine, lysine, isoleucine, and methionine. Methionine, in turn, is precursor for SAM synthesis. Aspartate is also involved in energy production under both aerobic and anaerobic conditions via tricarboxylic acid (TCA) cycle, gluconeogenesis, and fumarate respiration (*16*, *17*). Therefore, steady supply of aspartate is expected to be crucial for the growing cells.

In this study, we investigated the hypothesis that the Pcm enzyme is a major player in the aspartate salvage pathway in *Escherichia coli*. All in vitro characterizations of Pcm’s role in *E. coli* metabolism were performed using MG1655 strain. The 536 strain was used for investigating adaptation to urine and colonization of the mouse urinary tract. We found that Pcm plays a crucial role in the recycling of aspartate and is consequently involved in many aspects of bacterial metabolism. We demonstrated the biological relevance of the Pcm by showing that it contributes to the full growing capacity of cells under aerobic and anaerobic conditions. Last, we showed that Pcm facilitates adaptation to subinhibitory antibiotic doses and to the mammalian urinary tract during infection.

## RESULTS

### Maintenance of the intracellular aspartate pool in growing cells necessitates the involvement of the Pcm enzyme

Pcm-mediated conversion of IsoAsp into aspartate may contribute to aspartate recycling through protein degradation. This may be particularly important when aspartate availability via de novo synthesis or from growing medium is diminished. Dipeptides containing IsoAsp are degraded by isoaspartate dipeptidase A (IadA) (*18*), which is a widely distributed among bacteria. The activity of IadA, in addition to the general proteolysis machinery, allows for the degradation of IsoAsp-containing proteins to single amino acids. Therefore, both Pcm and IadA can contribute to the recycling of amino acids from IsoAsp-containing proteins.

**Fig. 1.**
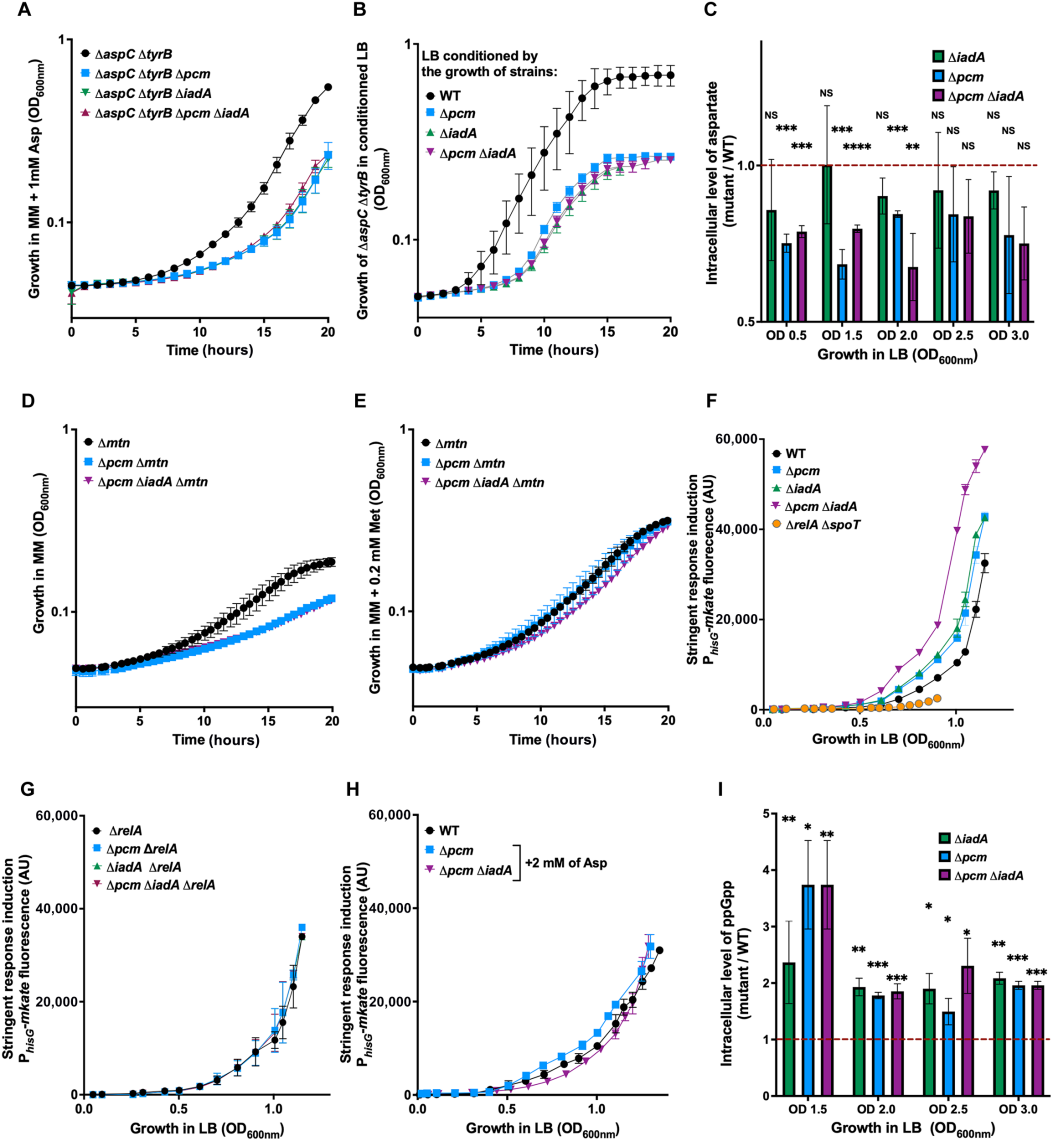
Impact of Pcm on the maintenance of intracellular aspartate level, on the stringent response induction and on the ppGpp level. (**A**) Growth of Δ*aspC* Δ*tyrB* strain, which is auxotroph for aspartate, and its Δ*pcm* and/or Δ*iadA* derivatives in minimal medium M9 supplemented with 0.4% glucose (MM) and 1 mM aspartate. (**B**) Growth of Δ*aspC* Δ*tyrB* strain in LB medium conditioned by the growth of WT, Δ*pcm*, Δ*iadA*, or Δ*pcm* Δ*iadA* strains*.* (**C**) Relative intracellular level of aspartate in Δ*pcm* and/or Δ*iadA* strains versus WT strain measured by LC-MS at different time points during growth in LB medium. (**D** and **E**) Growth of Δ*mtn* strain, which cannot recycle SAM, and its Δ*pcm* and Δ*iadA* derivatives in M9 minimal medium supplemented with 0.4% glucose (MM) (D) and 0.2 mM methionine (MM + 0.2 mM Met) (E). (**F**) Expression of the stringent response induction reporter P*_hisG_-mkate* during growth of WT, Δ*pcm*, Δ*iadA*, Δ*pcm* Δ*iadA*, and Δ*relA* Δ*spoT* strains in LB medium. (**G**) Impact of the *relA* gene deletion and of (**H**) the addition of aspartate 2 mM on the expression of the stringent response induction reporter P*_hisG_-mkate* during growth of WT and its Δ*pcm* and/or Δ*iadA* derivatives in LB medium*.* (**I**) Relative intracellular level of the ppGpp in Δ*pcm* and/or Δ*iadA* strains versus WT strain measured by LC-MS at different time points during growth in LB medium. (A, B, and D to H). Each point represents the mean value (±SD) from three independent experiments. (C and I) Each bar represents the mean value (±SD) from three independent experiments. Asterisks show significant differences compared to a fold change of 1 for each time point according to *t* test (*P < 0.05, **P < 0.01, ***P < 0.001, and ****P < 0.0001). All these experiments were performed using MG1655 strain. NS, not significant; AU, arbitrary units.

We first constructed Δ*aspC* Δ*tyrB* strain that is auxotrophic for aspartate, meaning that it cannot synthesize aspartate de novo and cannot grow in minimal medium without the addition of aspartate. We then deleted the *pcm* and/or *iadA* genes in this strain and monitored the growth of different mutants in M9 minimal medium supplemented with varying concentrations of aspartate. We found that when 1 mM aspartate was added to M9 medium, the growth rate of Δ*pcm*, Δ*iadA*, and Δ*pcm* Δ*iadA* derivatives was slower than that of *pcm*^+^*iadA*^+^ derivative (Fig. 1A). When aspartate concentration was less limiting, i.e., 2 mM, the growth of Δ*pcm* and Δ*iadA* derivatives was not affected, but Δ*pcm* Δ*iadA* derivative grew slower compared to *pcm*^+^*iadA*^+^ derivative (fig. S2A).

Next, we tested whether Pcm and IadA may be important for cell growth when aspartate availability from the growing medium is diminished. Because *pcm* and/or *iadA* mutant strains require higher concentrations of aspartate to grow as fast as the *pcm*^+^*iadA*^+^ strain, we thought that they might deplete aspartate faster from medium than wild-type (WT) strain. To test this hypothesis, we let WT and Δ*pcm* and/or Δ*iadA* mutant strains grow in LB medium until late exponential phase [optical density at 600 nm (OD_600nm_) = 2.5], after which bacterial cells were removed. Subsequently, we monitored growth of the WT and Δ*aspC* Δ*tyrB* strains in these different conditioned media. We found that Δ*aspC* Δ*tyrB* strain had extended lag time, i.e., the time needed to resume growth, in media conditioned by the growth all mutant strains compared to medium conditioned by the growth of the WT strain (Fig. 1B). A similar trend, albeit to a lesser extent, was observed for the growth of WT strain in different conditioned media (fig. S2B). Last, using liquid chromatography–mass spectrometry (LC-MS), we measured intracellular levels of aspartate of WT Δ*pcm*, Δ*iadA* and Δ*pcm* Δ*iadA* cells growing in LB medium. We found that Δ*pcm* and Δ*pcm* Δ*iadA* cells had lower levels of aspartate from that of WT until late exponential phase, while that of the Δ*iadA* was not different from WT (Fig. 1C). These results validated our hypothesis that Δ*pcm* mutant strain depletes aspartate from the LB medium faster than the WT strain.

### Pcm prevents an early induction of the stringent response

Aspartate serves as a precursor for the biosynthesis of threonine, lysine, isoleucine, and methionine (*19*). The rapid depletion of aspartate in the ∆*pcm* mutant strains suggests that the supply of these amino acids will also decrease during growth. To verify this hypothesis, we investigated the effect of deleting *pcm* and *iadA* genes on the methionine supply required for SAM de novo synthesis, which relies on the availability of aspartate. To achieve this, we deactivated the SAM recycling pathway, which provides two-thirds of the cellular SAM, by deleting *mtn* gene (*20*). We grew Δ*mtn*, Δ*mtn* Δ*pcm*, and Δ*mtn* Δ*pcm* Δ*iadA* strains in M9 minimal medium and observed that deleting *pcm* and *iadA* genes decreased the growth of the Δ*mtn* strain (Fig. 1D). However, adding methionine entirely restored the growth of the ∆*mtn* ∆*pcm* mutant and, to a great extent, that of the ∆*mtn* ∆*pcm* ∆*iadA* mutant, in comparison to the ∆*mtn* strain (Fig. 1E). These results demonstrate that accelerated aspartate depletion leads to a quicker decline in methionine availability.

When amino acids become limiting, uncharged tRNAs bind to the ribosome, signaling to the ribosome-associated RelA protein to synthesize ppGpp, which accumulation triggers the stringent response (*21*). We hypothesized that accelerated depletion of aspartate, and consequently of threonine, lysine, isoleucine, and methionine, would trigger the stringent response induction earlier in the ∆*pcm* and/or ∆*iadA* mutants than in the WT strain. To verify this hypothesis, we used a reporter fusion of the *hisG* gene promoter fused with *mkate* encoding fluorescent protein. The *hisG* gene codes for the enzyme responsible for the first step of histidine biosynthesis, whose expression is positively regulated by the cellular level of ppGpp (*22*). This reporter is a bona fide stringent response reporter, as it is not induced in the Δ*relA* Δ*spoT* (ppGpp^0^) strain (Fig. 1F).

By using this reporter, we observed that the stringent response is induced earlier and much more strongly during growth in cells lacking the *pcm* and/or *iadA* genes compared to WT cells (Fig. 1F). We deleted the *relA* gene in both the WT and mutant strains and observed that there was no difference between reporter inductions in different strains anymore (Fig. 1G). This confirms that the amino acid starvation was indeed responsible for the earlier and stronger reporter induction in mutant strains. Next, we verified whether the supplementation of growth medium with 2 mM aspartate would prevent the stringent response induction. As expected, the addition of aspartate erased the difference in reporter induction profile between the ∆*pcm* and Δ*pcm* Δ*iadA* mutant and WT cells, similar to the results observed upon deletion of the *relA* gene (Fig. 1H). Last, we corroborated results obtained using reporter fusion by quantifying ppGpp levels using LC-MS during growth of WT and different mutant cells. We observed up to 2.36-fold more ppGpp in Δ*iadA* cells and up to 3.61-fold more ppGpp in Δ*pcm* and Δ*pcm* Δ*iadA* cells than in WT. The increase in ppGpp level in mutant versus WT strain was the highest at the end of the exponential phase (OD_600nm_ = 1.5) (Fig. 1I). The correlation (*R*^2^ = 0.96, *P* < 0.0001; fig. S2C) between LC-MS measurements (Fig. 1I) and the measurements of P*_hisG_*-*mkate* fluorescence intensity (Fig. 1D) during growth further confirms that P*_hisG_*-*mkate* is an excellent reporter for intracellular ppGpp levels.

Together, these data show that Pcm contributes to the aspartate pool maintenance during growth thus allowing longer growth period and preventing an early induction of the stringent response. The involvement of the IadA enzyme may be due to the recycling of other amino acids from the IsoAsp-containing proteins.

### Pcm does not affect functioning of nongrowing cells but determines capacity of cells to resume growth

**Fig. 2.**
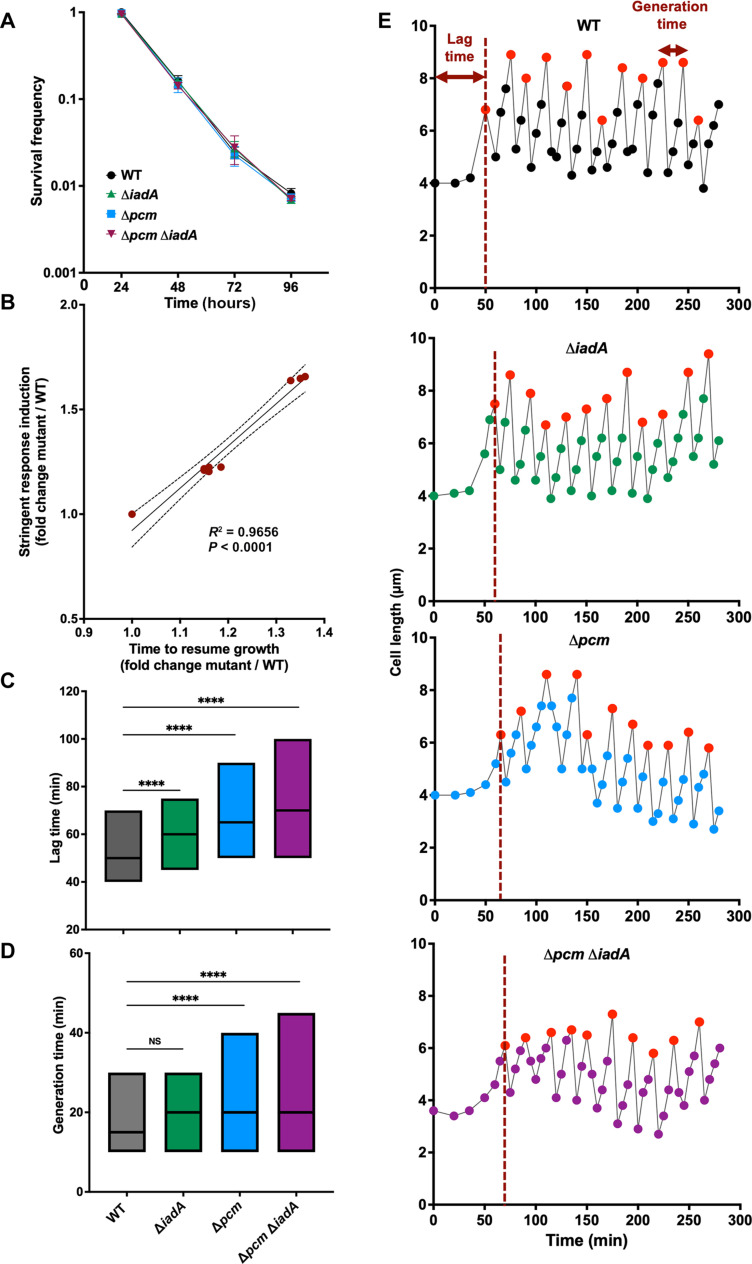
Impact of Pcm on the capacity of cells to survive and resume growth after treatment with bacteriostatic antibiotic and on the growth restart after stationary phase. (**A**) Survival of the WT, Δ*pcm*, Δ*iadA*, and Δ*pcm* Δ*iadA* strains treated with bacteriostatic antibiotic chloramphenicol (10 × MIC) during 96 hours. (**B**) Correlation between the fold increase in the maximal induction of the stringent response reporter P*_hisG_-mkate* and the fold increase in lag time duration after chloramphenicol treatment of mutant versus WT strains. (**C**) Time required for cells to start dividing after stationary phase, i.e., lag time, (**D**) and generation time calculated from the mother machine microfluidics experiments for at least 250 individual cells for each strain. For descriptive statistics, see table S3. Boxes represent the minimum and the maximum, and the midline indicates the median. Asterisks show significant difference of mutant compared to the WT according to Mann-Whitney test (*P < 0.05, **P < 0.01, ***P < 0.001, and ****P < 0.0001). (**E**) Growth of individual cells in the mother machine microfluidic device in LB medium. Each panel is a representative profile of growth of an individual WT, Δ*iadA*, Δ*pcm*, and Δ*pcm* Δ*iadA* mother cell. Cells were growing during 300 min. Lag time that is indicated by a vertical dashed line. Maximum cell length is marked by red dots (see fig. S3A). Generation time is defined as the time between cell birth and its division, corresponding to the time that separates two red dots. All these experiments were performed using MG1655 strain.

We showed that Pcm maintains steady supply of aspartate in growing cells. The question is whether Pcm contributes to functioning of the nongrowing cells. We treated cells with bacteriostatic antibiotic chloramphenicol [10× minimum inhibitory concentration (MIC)], which inhibits protein synthesis (*23*), and assessed cell viability as well as the time required for cells to resume growth after removal of the chloramphenicol, every 24 hours during 96-hour experiment. We observed a decline in cell viability over time, dropping eventually to ~1% for all strains, with no significant difference observed between different strains (Fig. 2A). In addition, we observed that the time needed to resume growth after removal of antibiotic increased with the incubation time for all strains. However, the increase was longer for the Δ*pcm*, Δ*iadA*, and Δ*pcm* Δ*iadA* strains compared to the WT strain (fig. S2C). Therefore, we observed significant positive correlation (*R*^2^ = 0.96, *P* < 0.0001) between the ratio in the time to resume growth of WT versus different mutant strains and the maximal induction of the stringent response reporter P*_hisG_-mkate* (Fig. 2B). This correlation supports the finding that the resumption of growth in *E. coli* cells follows the rule that the cells, which shut down their metabolism last at the beginning of stationary phase, are the first to respond to nutrients and start growing (*24*).

### Pcm is required for maximizing the cell growth potential

Next, we investigated the impact of the Pcm and IadA enzymes on the growth of individual cells in LB medium using a microfluidic “mother machine” device (Fig. 2, C to E; table S3; and fig. S3A) (*25*). The lag phase duration, i.e., the time to first division of a cell coming out of overnight stationary phase, was significantly longer for all mutant strains compared to the WT (Fig. 2C). The generation time, i.e., the time between two cell divisions, was significantly longer for Δ*pcm* and Δ*pcm* Δ*iadA*, but not for Δ*iadA*, compared to the WT (Fig. 2D). The maximal cell size was significantly smaller for Δ*pcm* and Δ*pcm* Δ*iadA*, but not for Δ*iadA*, compared to the WT (fig. S3A). The coefficient of variation in generation times was higher for Δ*pcm* and Δ*pcm* Δ*iadA* cells than for the WT, which was the same as that of the Δ*iadA* cells (table S3). Therefore, the loss of the *pcm* gene resulted in a loss of regularity in the cell cycle progression. Complementation of mutant strains with the functional *pcm* gene resulted in the complete restoration of Pcm^+^ phenotypes (table S3).

**Fig. 3.**
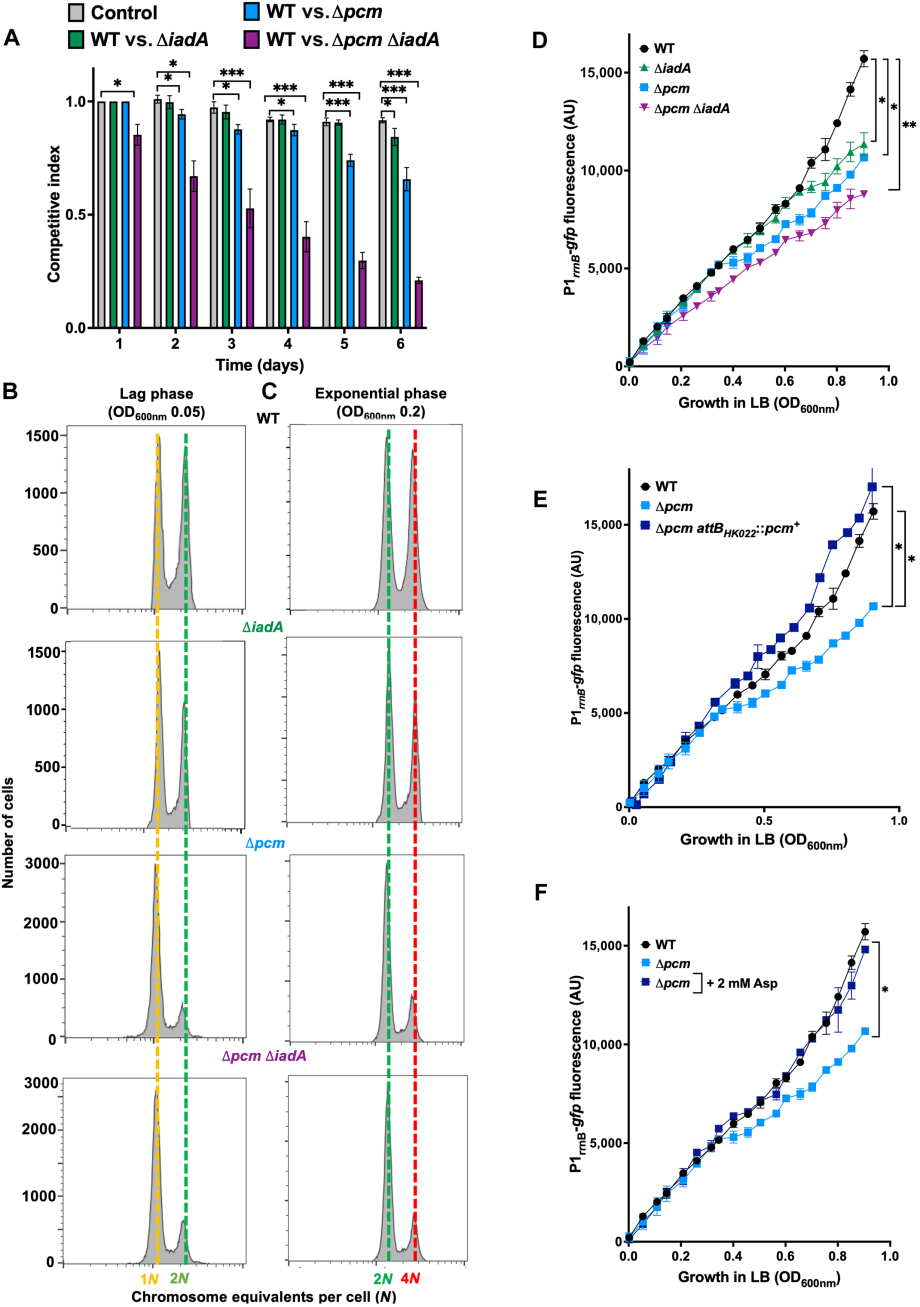
Impact of Pcm on fitness, DNA replication, and rRNA production. (**A**) Fitness of WT versus Δ*pcm*, Δ*iadA*, or Δ*pcm* Δ*iadA* strains measured by pairwise batch culture competitions during 6 days in LB medium*.* Fitness differences were estimated by calculating the competitive index (CI). To verify that neither of the two fluorescent reporters influences the competitive ability of the strain, a control competition assay Δ*pcm yfp* versus Δ*pcm mkate* was also performed. Bar graphs represent the mean value (±SD) of three independent experiments of CI analysis. Asterisks show significant differences compared to the control condition for each day according to *t* test (*P < 0.05 and ***P < 0.001). (**B** and **C**) DNA content in cells during lag phase (first column) (B) and cells growing exponentially (second column) (C) in LB medium. DNA content in WT, Δ*pcm*, Δ*iadA*, and Δ*pcm* Δ*iadA* cells was measured after 90 min of incubation with rifampicin and cephalexin. Number of genome equivalents (*N*) per cell was calculated using stationary phase, 1*N* cells, as a reference. The results are representative of three independent experiments. For statistical analysis, see fig. S3B. (**D** to **F**) Expression of the unstable *gfp*-based transcriptional reporter P1*_rrnB_*-*gfp*-asv, during exponential growth of WT, Δ*pcm*, Δ*iadA*, and Δ*pcm* Δ*iadA* strains in LB medium (D); WT, Δ*pcm*, and Δ*pcm attB_HK022_*::*pcm^+^* strains in LB medium (E);  WT and Δ*pcm* strain in LB medium supplemented or not with 2 mM aspartate (F). Each data point represents the mean value (±SD) from three independent experiments. Asterisks show significant difference compared to the WT according to *t* test (*P < 0.05 and **P < 0.01). All these experiments were performed using MG1655 strain.

Next, we measured the impact of the *pcm* and *iadA* gene deletions on fitness in 6-day batch culture competition experiments in LB medium. At the end of competition, after approximately 80 generations, all mutant strains had a significantly lower fitness than the WT strain (Fig. 3A). The fitness decrease in mutant strains was strongest for Δ*pcm* Δ*iadA* strain, followed by Δ*pcm*, while that of the Δ*iadA* strain was the weakest (Fig. 3A). Together, these results clearly show that Pcm and to lesser extent, IadA are required for maximizing the growth potential of *E. coli* cells.

### Pcm plays a critical role in the timing of DNA replication initiation

The successful progression through cell cycle requires coordination of the chromosome replication and cell division because it is imperative that chromosomes undergo proper replication and segregation before cell division. Thus, our observation of the positive impact of Pcm and IadA on the cell cycle prompted us to investigate whether they have also an effect on chromosome replication. We monitored the replication of chromosome in cells during lag and exponential growth phases, using a rifampicin and cephalexin run-out DNA replication assay (*26*). The distribution of DNA content per WT, Δ*pcm*, Δ*iadA*, and Δ*pcm* Δ*iadA* cells was in discrete narrow peaks that represent one, two, or four chromosomal equivalents per cell, which indicates the successful termination of all initiated chromosome replication cycles (Fig. 3, B and C). However, the populations of Δ*pcm* and Δ*pcm* Δ*iadA* cells exhibited a smaller proportion of cells having two chromosomal equivalents during lag phase compared to WT (Fig. 3B and fig. S3B). Inactivation of the *iadA* gene did not have significant impact on DNA replication (Fig. 3B and fig. S3B). The same trend was also observed in the populations of exponentially growing cells (Fig. 3C and fig. S3B). This suggests that the timing of initiation of chromosome replication was delayed in populations of the Δ*pcm* mutants compared to WT cells during both lag and exponential growth phases.

### Pcm contributes to the production of ribosomal RNA and energy production in growing cells

We showed that the Δ*pcm* and Δ*pcm* Δ*iadA* cells exhibit a longer lag and generation time, along with delayed initiation of chromosome replication. The duration of lag phase is known to be dependent on the time required for cells to attain high translational capacity, which is primarily achieved by increased production of ribosomal RNA (rRNA) (*27*–*29*). It is also known that the expression of rRNA operons correlates with cell growth rate (*30*, *31*). Therefore, we sought to investigate whether Pcm and IadA affect the rRNA production. For this, we used a fusion of the rRNA *rrnB* operon promoter P1 with the gene encoding an unstable green fluorescent protein (*32*, *33*). Our results showed that the expression level of the reporter was significantly lower in the Δ*pcm* and Δ*pcm* Δ*iadA* cells during exponential growth and, to a lesser extent, in the Δ*iadA* cells, when compared to the WT cells (Fig. 3D). Complementation of mutant strains with the functional *pcm* gene and supplementation of the LB medium with 2 mM aspartate resulted in the complete restoration of Pcm^+^ phenotypes (Fig. 3, E and F).

**Fig. 4.**
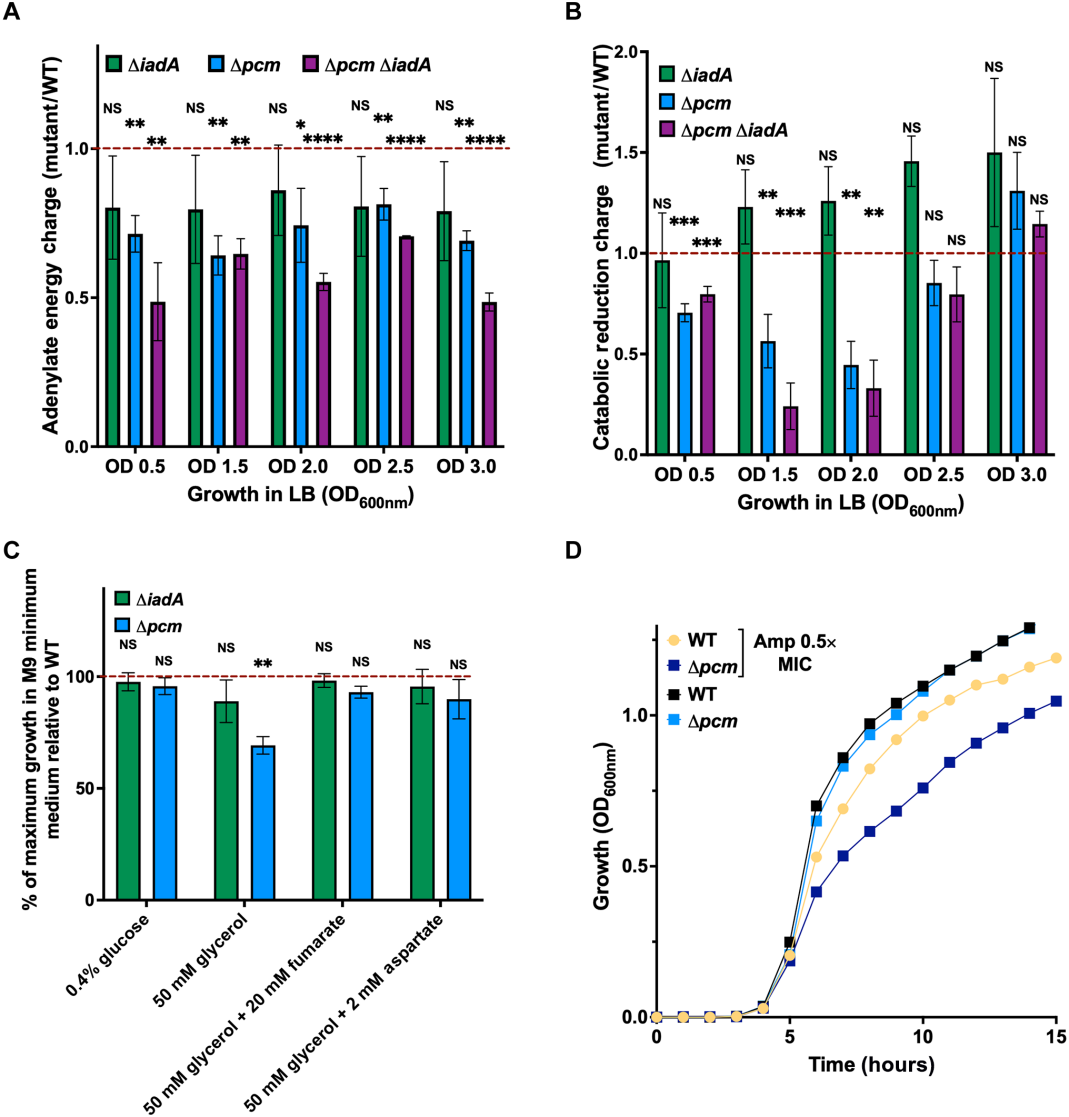
Impact of Pcm on energy state under aerobic and anaerobic conditions and on growth in presence of subinhibitory concentration of antibiotic. (**A**) AEC fold change between each mutant and WT cells at different time points during growth in LB medium. The fold changes between each mutant and WT cells were calculated from the LC-MS measurement of intracellular relative level of ATP, ADP, and AMP (see fig. S3, D to F). (**B**) CRC fold change between each mutant and WT cells at different time points during growth. The fold changes between each mutant and WT cells were calculated from the LC-MS measurement of intracellular relative level of NAD^+^ and NADH. (A and B) Each bar graph represents the mean value (±SD) from three independent experiments. Asterisks show significant differences compared to a fold change of 1 for each growth time point according to *t* test (*P < 0.05, **P < 0.01, ***P < 0.001, and ****P < 0.0001). (**C**) Maximum density of WT, Δ*pcm*, Δ*iadA*, and Δ*pcm* Δ*iadA* bacterial populations measured by spectrophotometer at OD_600nm_ after 24-hour growth under anaerobic conditions. Strains were grown in M9 medium supplemented with glucose or glycerol (with or without fumarate and aspartate). The results are expressed as percentage of mutant versus WT (corresponding to 100%) growth for each condition. Each bar graph represents the mean value (±SD) from three independent experiments. Asterisks show significant differences compared to the WT for appropriate growth conditions according to Mann-Whitney test (**P < 0.01). (**D**) Growth of WT and Δ*pcm* in LB medium with or without 0.5 MIC of ampicillin (Amp). The results are representative of three independent experiments. All these experiments were performed using MG1655 strain.

Because it is also well established that the rRNA promoter activity is dependent on the energy level in the cells, i.e., ATP and GTP (*32*), we decided to evaluate the impact of Pcm and IadA on energy production. To achieve this, we quantified ATP, adenosine 5′-diphosphate (ADP), adenosine 5′-monophosphate (AMP), and GTP by LC-MS in WT, Δ*pcm*, Δ*iadA*, and Δ*pcm* Δ*iadA* cells during different growth phases in LB medium (fig. S3, D to G). The GTP concentration was lower in all mutant strains compared to WT (fig. S3G). We calculated the adenylate energy charge (AEC), which measures the amount of metabolic energy transiently present in the adenine nucleotide pool (*34*). We found that the AECs of Δ*pcm* and Δ*pcm* Δ*iadA* strains, but not that of the Δ*iadA* strain, were significantly lower than that of the WT strain (Fig. 4A). We also evaluated ATP concentration in individual cells, which were growing in the LB medium, using ratiometric fluorescent ATP indicator “QUEEN2M” (*35*). We found again that Δ*pcm* and Δ*pcm* Δ*iadA* cells, but not Δ*iadA* cells, have lower ATP concentration than WT (fig. S3H).

We also investigated whether supplementing the growth medium with molecules that contribute to energy production through the TCA cycle could restore Pcm^+^ phenotypes. We tested how aspartate-derived amino acids (methionine, isoleucine, lysine, and threonine), which can be converted into TCA cycle intermediates, as well as citrate, succinate, malate, and fumarate, affect expression of the P*_hisG_-mkate* and P1*_rrnB_-gfp* reporters (figs. S4 and S5). We found that only aspartate was able to completely restore Pcm^+^ phenotypes to the *pcm* mutant.

Previous studies have demonstrated that the proper functioning of chaperones and proteases responsible for maintaining protein homeostasis relies heavily on energy, particularly ATP. Consistent with this feature, these studies have revealed that cells in the stationary phase tend to accumulate protein aggregates. This accumulation occurs as a consequence of disruptions in protein homeostasis caused by the decline in ATP levels, which, in turn, impairs the capacity of chaperones and proteases to effectively clear these protein aggregates (*36*). Hence, we investigated whether the reduced cellular energy level we uncovered in the absence of *pcm* is not a result of increased level of protein aggregation stemming from IsoAsp accumulation, both during the exponential and the stationary phases. The accumulation of misfolded or aggregated proteins serves as a trigger for the induction of the heat shock response, a conserved protective mechanism that safeguards cellular protein homeostasis. Activation of the heat shock response primarily entails the tightly regulated production of various molecular chaperone proteins and protease enzymes. Among these proteins, inclusion body protein A (IbpA), a small molecular chaperone, whose gene is one of the most strongly induced during the heat shock response (*37*, *38*), exhibits a strong affinity for binding to protein aggregates (*39*). Therefore, to assess the impact of *pcm* deletion on the induction of the heat shock response and on protein aggregate levels, we used transcriptional P*_ibpA_*-*yfp* and translational IbpA–yellow fluorescent protein (YFP) reporters. We monitored the induction of the P*_ibpA_*-*yfp* reporter at various time points during the growth and observed no significant differences between the WT and Δ*pcm* strains (fig. S6A). Next, we monitored protein aggregation in growing and stationary phase cells for 96 hours. We did not observe significant difference between different strains in the number of cells with at least one IbpA-YFP focus or in the fluorescence intensity of individual fluorescent foci (fig. S6, B to E). These results show that the deletion of the *pcm* gene does not result in high level of protein aggregation.

We also quantified the oxidized and reduced forms of NAD, i.e., NAD^+^ and reduced form of NAD^+^ (NADH), respectively, by LC-MS. NAD^+^ and NADH are major players in metabolism because they are cofactors for over 300 redox reactions (*40*). As NADH fuels ATP synthesis via the aerobic respiratory catabolism, relative levels of NAD^+^ and NADH are critical indicators of the energy state. We calculated the catabolic reduction charge (CRC) (*41*), which measures general intracellular redox potential and hence metabolic state. We found that, until late exponential phase, the CRCs of Δ*pcm* and Δ*pcm* Δ*iadA* strains, but not that of the Δ*iadA* strain, were significantly lower than that of the WT strain (Fig. 4B).

As aspartate serves as a major source of energy through fumarate respiration in *E. coli* under anaerobic conditions (*42*), we monitored the growth of WT, Δ*pcm*, and Δ*iadA* strains in M9 medium supplemented with glycerol under anaerobic conditions. Under these conditions, where aspartate is the substrate of fumarate respiration, the maximal density of Δ*pcm* strain was significantly reduced compared to the WT strain, while that of the Δ*iadA* strain was similar to the WT strain (Fig. 4C). The addition of fumarate or 2 mM aspartate improved the growth of Δ*pcm* mutant strain (Fig. 4C). In summary, our results show that Pcm is required for the maximal production of rRNA, which is required for full translational capacity, and for energy production in growing cells.

### Pcm and IadA are required for maximizing the growth of cells treated with sublethal doses of ampicillin

We previously reported that when *E. coli* cells are exposed to sublethal doses of ampicillin, they increase energy production and translation capability, which allows for both efficient antibiotic-induced damage control and maintenance of the rapid growth (*43*). However, they deplete nutrients from the growth medium, including aspartate, and trigger stringent response faster than untreated bacteria. In this study, we examined whether the loss of the *pcm* and/or *iadA* genes may aggravate impact of the sublethal dose of ampicillin on cell growth. Our results showed that all mutant strains had slower growth rates and lower maximal density levels compared to the WT strain (Fig. 4D and fig. S6, F and G). Therefore, our data show that the Pcm and IadA enzymes enhance the robustness of bacteria exposed to sublethal antibiotic treatments presumably by maintaining the intracellular aspartate pool and by recycling other amino acids from the IsoAsp-containing proteins, respectively.

### Pcm plays a crucial role in the ability of *E. coli* to colonize urinary tract

Most strains of *E. coli* species are harmless commensals of the gastrointestinal tract of humans and warm-blooded animals (*44*). However, some strains have pathogenic potential (*45*). For example, uropathogenic *E. coli* (UPEC) strains are responsible for 80% to 90% of urinary tract infections (UTIs) in humans (*46*). UPEC strains primarily colonize the nutritionally diverse environment of the host intestinal tract but can invade the nutritionally poorer urinary tract (*47*, *48*). During UTI, UPEC strains rely on amino acids and small peptides from the urine as carbon sources (*46*–*49*). Urine contains a low concentration of aspartate, primarily in peptides (*50*). IsoAsp-containing peptides have also been detected in urine (*51*). Moreover, it was reported that the *pcm* gene is induced in UPEC CFT073 strain during infection of mouse urinary tract (*52*). Given these observations and our findings, we hypothesized that Pcm may play a crucial role in the metabolic adaptation and colonization ability of *E. coli* in the urinary tract.

**Fig. 5.**
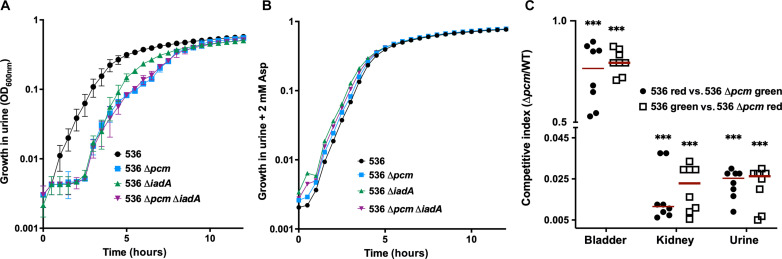
Impact of Pcm on growth of *E. coli* 536 strain in urine and on the colonization of mouse urinary tract. (**A** and **B**) Growth of the 536 strain and its Δ*pcm*, Δ*iadA*, and Δ*pcm* Δ*iadA* derivatives in human urine (A) supplemented with 2 mM aspartate (B). Each data point represents the mean value (±SD) from three independent experiments. (**C**) Competitions between 536 *attB*λ::P*_lac_-mkate* versus 536 Δ*pcm attB*λ::P*_lac_-gfp* and 536 *attB*λ::P*_lac_*-*gfp* versus 536 Δ*pcm attB*λ::P*_lac_*-*mkate* strains. Fitness differences in the bladder, the kidney, and urine were estimated by calculating the CI 48 hours after infection. Red lines indicate the median of two independent experiments with eight mice for each competition assay. Asterisks show significant differences compared to the WT according to Mann-Whitney test (***P < 0.001). All these experiments were performed using 536 strain.

We decided to use model UPEC strain 536 to investigate the role of Pcm during UTI in the mouse model. We first confirmed that Pcm plays a critical role in the timing of DNA replication initiation (fig. S4, B and C) and energy production (fig. S7D), thus contributing to cell fitness both in 536 as in the MG1655 strains (Fig. 3A and fig. S7A). Next, we monitored the growth of 536 and its 536 Δ*pcm*, 536 Δ*iadA*, and 536 Δ*pcm* Δ*iadA* derivatives in human urine, and we observed that growth of mutant strains was more affected than that of the 536 strain (Fig. 5A). We did not detect any differences in growth among these strains in urine supplemented with 2 mM aspartate (Fig. 5B). Last, we investigated the role of Pcm in UPEC strain ability to colonize mouse urinary tract by performing competitions between 536 and its 536 Δ*pcm* derivative. Two days after infection, we measured the relative ability of the two strains to colonize the urine, bladder, and kidneys. We observed a significant reduction in the competitive ability of the 536 Δ*pcm* mutant in the bladder, and a 100 × reduction was observed in the urine and kidneys (Fig. 5C). Therefore, our data show that Pcm plays a crucial role in the ability of *E. coli* to colonize urinary tract. This observation confirms that besides virulence factors, metabolic capacity is important for the pathogenicity (*53*, *54*).

## DISCUSSION

The evolutionary conservation and wide distribution in all domains of life of the Pcm enzyme, which is crucial for IsoAsp repair, indicate that this form of amino acid damages is deleterious and occurs at high frequency. In this study, we showed the highly pleiotropic extent of Pcm’s involvement in the proper functioning of the growing *E. coli* cells. We showed that Pcm is required for the energy production (Fig. 4A and fig. S3, D to G), as well as for the maintenance of cellular redox potential (Fig. 4B) and the production of SAM (Fig. 1, D and E), which are critical for the proper functioning of the majority of metabolic pathways. We demonstrated the biological relevance of the Pcm enzyme by showing that it contributes to the full growing capacity of cells under aerobic and anaerobic conditions (Figs. 2, C to E; 3A; and 4C and table S2), cells exposed to subinhibitory antibiotic doses (Fig. 4D and fig. S6, F and G), and during urinary tract colonization (Fig. 5C). Although Pcm activity contributes to the timing of when cells stop growing and enter stationary phase (Fig. 1, F to I), it has no detectable impact on the functioning of nongrowing cells (Fig. 2A and fig. S2D). The fact that expression of the *E. coli pcm* gene is regulated by the vegetative sigma factor RpoD, and not by stationary phase sigma factor RpoS (*55*), further supports idea that Pcm is important primarily for growing cells.

The deletion of the *iadA* gene has a different impact on cellular functions compared to that of the *pcm* gene. The deletion of *iadA*, unlike the deletion of *pcm*, has no discernible impact on various vital processes such as energy production, redox potential maintenance, SAM production, or DNA replication, all of which rely on aspartate and its derivatives. This is consistent with the fact that the contribution of IadA to Asp salvage by degrading IsoAsp-containing dipeptides is not significant in Pcm-proficient cells (Fig. 1C). The phenotypes of IadA-deficient cells can be explained by their inability to recycle other amino acids present in IsoAsp-containing peptides. However, many phenotypes of the Pcm-deficient cells are accentuated by deletion of the *iadA* gene.

We showed that the loss of the *pcm* gene leads to faster depletion of aspartate from growth medium (Fig. 1, B and C), causing reduced expression of gene coding for rRNA, which is sensitive to the cellular energy state (Fig. 3D), slower growth (Figs. 1, A and B; and 2, C to E; and table S3) and early induction of the stringent response (Fig. 1A). Adding aspartate to the medium can reverse all these phenotypes (Figs. 1H and 3H and fig. S2A), while adding all aspartate-derived amino acids, i.e., methionine, isoleucine, lysine, and threonine, cannot (figs. S4C and S5C). Likewise, adding of the TCA cycle intermediates (citrate, succinate, malate, and fumarate) as an additional energy source did not reverse these phenotypes (figs. S4, D to G, and S5, D to G). We also showed that inactivation of the *pcm* gene diminishes growth of *E. coli* in human urine (Fig. 5A), which contains very low levels of aspartate, while supplementing the urine with aspartate restores growth of the *pcm* mutant to the WT level (Fig. 5B).

The question that arises from these observations pertains to the cause-consequence relationship between the loss of Pcm function and aspartate depletion: Do Pcm-associated phenotypes result from the involvement of Pcm in protein repair or from some other Pcm functions? Although many articles suggest that Pcm is primarily protein repair enzyme (*6*, *12*, *13*, *56*, *57*), several lines of evidence indicate otherwise. First, IsoAsp can form in both proteins and peptides (*58*), and Pcm is more efficient in processing IsoAsp on peptides than on proteins (*14*). Second, Pcm’s action on IsoAsp, whether derived from aspartate or asparagine, always results in the production of aspartate (*15*). This leads to asparagine-to-aspartate substitutions in proteins, potentially affecting their function [for an example of the deleterious impact of asparagine-to-aspartate substitution on protein function, see (*59*)]. Because Pcm exhibits such high error-prone activity, it seems unlikely that its primary biological function is protein repair. Third, if aspartate and asparagine are transformed to IsoAsp in only a very small fraction of proteins, then that may not significantly affect overall functioning of pool these proteins unless those proteins that are present in very small copy number per cell. However, mutated and damaged proteins, regardless of their functions, tend to aggregate. Therefore, if Pcm is a protein repair enzyme, then the loss of Pcm function is expected to increase protein aggregation. However, we observed no difference in either number or size of protein aggregates between WT and *pcm* mutant cells during exponential growth or stationary phase. Together, this information supports the hypothesis that Pcm is not primarily a protein repair enzyme.

**Fig. 6.**
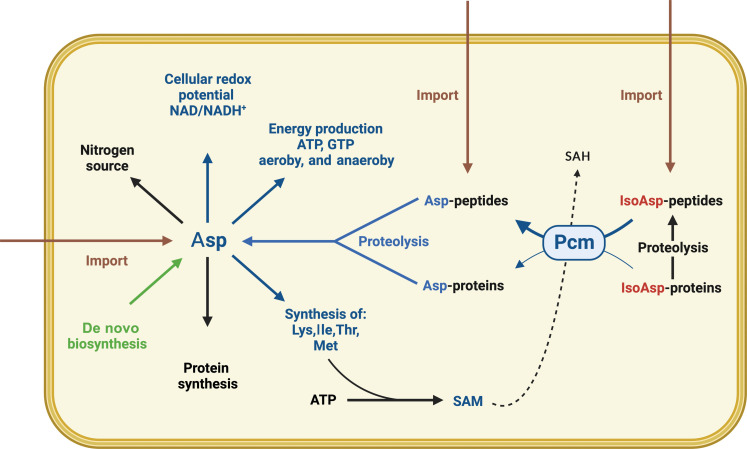
Role of Pcm-dependent aspartate salvage pathway in *E. coli* metabolism. Aspartate (Asp) plays a pivotal role in metabolism of growing cells because besides being proteinogenic amino acid, it is also a precursor for the biosynthesis of threonine (Thr), lysine (Lys), isoleucine (Ile), and methionine (Met). Met, in turn, serves as a precursor for the synthesis of SAM. Asp is involved in energy production under both aerobic and anaerobic conditions. Asp contributes to the maintenance of cellular redox potential NAD/NADH^+^ and serves as an important nitrogen source. Asp is provided by de novo synthesis (green arrow) and import from the environment (brown arrows). Asp can be imported in the form of free amino acids or in peptides containing Asp and IsoAsp (brown arrows). Pcm salvages Asp from IsoAsp-containing peptides (blue arrows). SAH, *S*-adenosylhomocysteine.

We propose that the salvaging of intracellular aspartate and the scavenging of extracellular aspartate from IsoAsp-containing peptides catalyzed by *E. coli* Pcm enzyme play a crucial role in satisfying the overall aspartate biosynthesis needs (Fig. 6). This is especially significant in environments where the primary source of aspartate is the catabolism of environmental peptides, some of which contain IsoAsp. These conditions are observed when *E. coli* grows in complex media, such as LB, and in natural environments such as the host urinary tract (*50*, *51*, *60*–*62*). For instance, in human urine, the Pcm-mediated aspartate salvaged from IsoAsp would increase the amount of aspartate available for cellular functioning by up to 40% (*51*, *62*). The evolutionary conservation of the Pcm enzyme raises the intriguing possibility that eukaryotic cells may also harbor a Pcm-dependent aspartate-salvaging pathway, which could contribute to cellular functioning.

## MATERIALS AND METHODS

### Strains, media, culture conditions, and chemicals

Bacterial strains and plasmids used in this study are described in table S1. All strains are derivatives of two *E. coli* strains: the K12 MG1655 commensal strain (*63*) and the uropathogenic 536 (O6:K15:H31) strain isolated from a patient with acute pyelonephritis (*64*). All in vitro characterizations of the Pcm and IadA roles in *E. coli* metabolism were performed using MG1655 strain. The 536 strain was used for investigating adaptation to urine and colonization of the mouse urinary tract.

All the experiments were performed at 37°C unless mentioned otherwise, using LB medium or M9 minimal salts (Serva) supplemented with 0.4% glucose or with 50 mM glycerol as a carbon source and complemented or not with 2 mM isoleucine, 2 mM leucine, 2 mM threonine, and 2 mM methionine or with 0.4 mM tyrosine or with different concentrations of aspartate (ranging from 0.2 to 2 mM). When needed, the medium was supplemented with antibiotics (Sigma-Aldrich) at the following concentrations: ampicillin (100 μg ml^−1^), chloramphenicol (30 μg ml^−1^), and kanamycin (100 μg ml^−1^). When needed, the medium was supplemented with isopropyl-β-d-thiogalactopyranoside (IPTG) at 0.2 mM to induce P*_lac_*-*gfp* and P*_lac_*-*mkate.*

### Construction of MG1655 strain derivatives

All derivatives of *E. coli* K12 MG1655, which is referred to as WT, were constructed by P1vir transduction of alleles from either the Keio (*65*) or from the laboratory collection. To remove the antibiotic resistance gene flanked by flipase recognition target sequences from the chromosome, the derivatives were transformed with plasmid pCP20 coding for the flipase. The resulting transformants were then treated as previously described (*66*).

To be sure that the phenotypes observed for Δ*pcm* mutant strains were due to the deletion of *pcm* gene, the Δ*pcm* mutant strains were complemented with a chromosomal copy of the functional *pcm* gene under the control of its native promoter. The native promoter and the gene were amplified from the WT chromosome and integrated in the phage *attB_HK022_* site using already described one-step cloning and chromosomal integration pOSIP plasmids (*67*). The primers used are listed in table S2.

### Construction of 536 strain derivatives

*pcm* and *iadA* genes were deleted from the chromosome of *E. coli* 536 using gene replacement methods based on the λ red recombinase system (*66*). A recombinogenic fragment consisting of a removable chloramphenicol resistance gene from pKD3 flanked by 40–base pair sequences homologous to the upstream and downstream extremity of the targeted region that was to replace was amplified by polymerase chain reaction (PCR). The primers used are listed in table S2. Strains carrying the temperature-sensitive plasmid pKD46 coding the λ red recombinase system were treated as described previously (*66*). For this process, terrific broth medium was used. Purified recombinant clones were verified by PCR. The antibiotic resistance gene was removed using pCP20 plasmid (*66*).

The 536 and derivatives were labeled with a chromosomal inducible fluorescent reporter genes, i.e., *gfpmut2* or *mkate*, that are under the control of the inducible promoter PL*_lacO1_*, which were integrated in the in the lambda phage integration site *attB* (*attB*_λ_) on the chromosome as previously described (*68*). The lactose promoter pL*_lacO1_* was amplified from the template plasmid pZE12luc (Expressys). *gfpmut2* and *mkate* genes were amplified from the pUA66 plasmid (*69*) or from the laboratory collection.

### Determination of LB medium depletion in aspartate

To estimate the LB medium depletion in aspartate generated by the growth of WT, Δ*pcm*, Δ*iadA*, or Δ*pcm* Δ*iadA* mutant cells, we used conditioned LB medium and a strain auxotroph for aspartate (Δ*aspC* Δ*tyrB*). Conditioned LB medium is defined here as LB medium that has sustained the growth of one of these strains until an OD_600nm_ of 2.5. The corresponding spent medium was centrifuged, and the supernatant was then filtered using a 0.2-μm filter unit. Then, we monitored the growth of Δ*aspC* Δ*tyrB* strain in the different conditioned LB medium*.* In this experimental setup, cell growth can be sustained only by aspartate that is left in the medium. Therefore, we can estimate the specific medium depletion in aspartate caused by the growth of mutant cells and compared to the one caused by WT strain. Overnight cultures of Δ*aspC* Δ*tyrB* were diluted 1:1000 and dispensed in 96-well plates. In each well, the volume of the culture was 150 μl of conditioned LB medium and 50 μl of mineral oil. During 20 hours of growth at 37°C with shaking, OD_600nm_ was measured every 5 min using a BioTek Synergy H1 microplate reader.

### Determination of growth curves

Overnight cultures were diluted 1:1000 and dispensed in 96-well plates; in each well, the final volume was 150 μl of LB or M9 minimal medium and 50 μl of mineral oil. It has been previously found that the addition of mineral oil does not affect bacterial growth and does not provoke the expression of genes induced during anaerobic growth (*69*). During 20 hours of growth at 37°C with shaking, OD_600nm_ was measured every 5 min using a BioTek Synergy H1 microplate reader.

### Measuring expression of fluorescent protein–based transcription reporter

Overnight cultures were diluted 1:1000 and dispensed in 96-well plates. In each well, the final volume was 150 μl of LB or M9 minimal medium and 50 μl of mineral oil. During 20 hours of growth at 37°C with shaking, OD_600nm_ was measured every 5 min. For *gfp*-based reporter, the Synergy H1 microplate reader was used with excitation at 485 nm and emission at 510 nm for analysis. For *mkate-*based reporter, the Tecan Spark microplate reader was used with excitation at 590 nm and emission at 630 nm for analysis.

### Determining duration of the recovery phase after antibiotic treatment

Overnight cultures were diluted 1:1000 in fresh LB medium and incubated at 37°C with agitation until OD_600nm_ reached 0.2. Then, cells were diluted 1:1000 in fresh LB medium and grown again until they reached an OD_600nm_ of 0.6. The bacteriostatic antibiotic chloramphenicol was added at a concentration of 18 μg ml^−1^ corresponding to 10× MIC. Chloramphenicol inhibits protein synthesis but does not impact metabolic activity controlled by already synthesized proteins, thus mimicking quiescence cells’ state. After 48 hours of treatment, chloramphenicol (6 μg ml^−1^) was added every 24 hours to ensure the antibiotic pressure maintenance. Every 24-hours cultures were sampled, and cells were washed three times in LB medium. Cell viability was assessed by colony-forming units counting, as well as the time required for cells to resume growth by monitoring growth in fresh LB medium in 96-well plates.

### Microfluidics experiments

Cells from overnight cultures were loaded into a microfluidic mother machine device (*25*) by diffusion. The mother machine microfluidic device consists of separate growth channels, which are closed on one end and open to the medium channel on other. The “mother” cell is retained at the closed end, while sister and daughter cells are pushed along the growth channel toward the medium channel until they are washed away.

Fresh LB medium was infused with a syringe pump at 1 ml hour^−1^. For each experiment, images were acquired for 5 hours at 5-min intervals using MetaMorph software and a Nikon Eclipse Ti fluorescence microscope equipped with a motorized stage and a charge-coupled device camera (Photometrics CoolSnap HQ2). To extract information about the cells from the images, each channel containing cells was cut and pasted on a new image and aligned from left to right according to the acquisition time. The resulting image contained cell segmentation information and was analyzed by ImageJ. The cell lineage was determined by visualizing the arrows connecting the cells. The division time of one cell was defined as the time between its birth and its division (*29*).

### Competition in batch cultures

We determined the relative fitness of MG1655 and 536 Δ*iadA*, Δ*pcm*, and Δ*pcm* Δ*iadA* mutants compared to the WT and 536 cells, respectively, in LB medium, supplemented with 20 mM IPTG for *E. coli* 536 strains. Overnight cultures of the different strains were diluted 1:10,000 in fresh LB medium mixed at 1:1 final ratio. Every 24 hours, cultures were diluted in fresh medium. Competition cycles were repeated for 6 days. To determine the relative amount of each strain, samples were taken after each competition cycle. The competing strains were differentiated on the basis of different fluorescence. MG1655 and its mutants carried a chromosomic constitutive transcription fluorescent reporter, either PR*yfp* or PR*mkate* integrated in the phage site *intC.* The *E. coli* 536 and its mutants carried a chromosomal inducible fluorescent reporter, either PL*_lacO1_*-*gfp* or PL*_lacO1_*-*mkate* integrated in the *attB*_λ_ site*.* To verify that the two fluorescent reporters have no differential impact on competitive ability, each mutant strain carrying *gfp* and *mkate* fluorescent reporters were also put in competition. The relative proportion of each fluorescence, i.e., WT or mutant strain, was detected using Beckman Coulter Gallios flow cytometer with a maximum of 2000 events counted per second and 50,000 cells analyzed. Relative fitness of the mutant strain was estimated by calculating the competitive index (CI): ${\mathrm{CI}}_{n}=\frac{{\text{Day}}_{n}\left(\%\frac{\text{mutant}}{\text{WT}}\right)}{{\text{Day}}_{0}\left(\%\text{mutant}/\text{WT}\right)}$

### DNA content analysis

DNA content quantification was performed as previously described (*26*). Overnight cultures were diluted 1:1000 in fresh LB medium and incubated at 37°C with agitation until OD_600nm_ reached 0.05 (considered as lag phase) and OD_600nm_ reached 0.2 (considered as log phase). Cephalexin (10 μg ml^−1^) and rifampicin (150 μg ml^−1^) were added simultaneously to respectively block cell division and inhibit newly initiation of replication cycle. Rifampicin inhibits initiation of replication but allows ongoing replication rounds to finish, thus enabling estimation of the number of chromosome equivalents. Cells were incubated simultaneously with both antibiotics at 37°C for an additional 90 min. Cells were washed with 10^−2^ M MgSO_4_ before being fixed with 100% cold methanol for 1 hour. Fixed cells were washed with phosphate-buffered saline, and the DNA was stained with DAPI (4′,6-diamidino-2-phenylindole; 0.3 μg ml^−1^). For each time point, a minimum of 50,000 cells was analyzed using Beckman Coulter Gallios flow cytometer with a maximum of 2000 events counted per second. DAPI was excited with an ultraviolet laser at 355 nm and read at 450 nm. The number of the peaks of histograms showing amount of DNA in these cells corresponds to the number of fully replicated chromosomes and reflects the number of origins present in the cell at the time the drugs were added. Number of genome equivalents (*N*) was calculated using stationary phase cells (1*N*) as a reference.

### LC-MS measurements of ATP-, ADP-, AMP GTP–, and ppGpp-relative quantification

WT Δ*pcm*, Δ*iadA*, and Δ*pcm* Δ*iadA* overnight cultures were diluted 1:1000 in fresh LB medium and incubated at 37°C with agitation. At different time points, approximately 10^10^ cells were harvested. ATP-, ADP-, AMP GTP–, NAD^+^-, NADH-, and ppGpp-relative quantification was performed by LC-MS as previously described (*64*, *65*). Briefly, metabolic activity was blocked by immersion in liquid nitrogen for 10 s. Metabolites were extracted using a solvent mixture of methanol/acetonitrile/H_2_O (50:30:20) at −20°C (extraction solution). Samples were vortexed for 5 min at 4°C and then centrifuged at 16,000*g* for 15 min at 4°C.

The supernatants were collected and analyzed by LC-MS using SeQuant ZIC-pHilic column (Millipore) for the LC separation. The peak areas, corresponding to relative measurements of different metabolites, were determined (*70*, *71*). For each time point, three biological replicates were performed. The results are expressed as relative measurements ratio between each mutant and WT cells. Each calculated ratio indicates the fold change in the intracellular metabolite relative concentration in mutant cells compared to the WT cells. From the fold change of ATP-, ADP-, and AMP-relative intracellular concentration, we calculated the AEC fold change using the Atkinson equation (*34*): $\text{AEC}=\frac{\left[\text{ATP}\right]+\frac{1}{2}\left[\text{ADP}\right]}{\left[\text{ATP}\right]+\left[\text{ADP}\right]+\left[\text{AMP}\right]}$ . From the fold change of NAD^+^- and NADH-relative intracellular concentration we calculated the CRC using the Andersen equation (*41*): $\text{CRC}=\frac{\left[\text{NADH}\right]}{\left[\text{NADH}\right]+\left[\text{NAD}\right]}$.

### Single-cell ATP measurements

ATP level was estimated in living *E. coli* MG1655 and *E. coli* 536 cells at different time points during growth in LB medium. Overnight cultures carrying the previously described QUEEN-2M ATP biosensor plasmid pWL2 (*35*) were diluted 1:1000 in fresh LB medium supplemented with 60 mM IPTG to induce the synthesis of the QUEEN-2M biosensor and incubated at 25°C with agitation until OD_600nm_ reached 0.2 (considered as early exponential phase) and OD_600nm_ reached 0.5 (considered as middle exponential phase). For each time point, cultures were sampled and a minimum of 50,000 cells was analyzed using Beckman Coulter Gallios flow cytometer with a maximum of 2000 events counted per second. QUEEN-2M is a ratiometric biosensor that has excitation peaks at 405 and 488 nm corresponding to the ATP-bound and ATP-free forms, respectively. Samples were excited at both 405 and 488 nm and read at 513 nm. We calculated ratio of fluorescent intensities between the two spectra, denoted as *R*_ATP(405ex/488ex)_, which acts as a proxy for the intracellular ATP level (*35*).

### Determination of the heat shock response induction using P*_ibpA_*-*yfp* reporter

The induction of the heat shock response was monitored during cell growth using the previously described P*_ibpA_-yfp* transcriptional reporter (*39*). WT and Δ*pcm* overnight cultures were diluted 1:1000 in fresh LB medium and incubated at 37°C with agitation. At different time points, cultures were sampled, and a minimum of 50,000 cells was analyzed using Beckman Coulter Gallios flow cytometer with a maximum of 2000 events counted per second. Samples were excited at 488 nm and read at 530 nm.

### Protein aggregation detection using IbpA-YFP reporter

The level of protein aggregation in the cells was scored using the previously described fluorescent IbpA-YFP translational reporter (*39*). Overnight cultures were diluted 1:1000 and incubated at 37°C until 24, 48, 72, or 96 hours of stationary phase. At each time point, cultures were sampled, cells were examined with a 100× objective, and images were acquired using MetaMorph software and a Nikon Eclipse Ti fluorescence microscope equipped with a charge-coupled device camera (Photometrics Coolsnap HQ2). The level of protein aggregation in the cells was estimated by quantifying the percentage of cells with at least one IbpA-YFP focus and the intensity of fluorescence associated with each individual IbpA-YFP focus.

### Determination of growth capacity under anaerobic conditions

WT Δ*pcm* and Δ*iadA* overnight cultures aerobically grown in M9 minimal medium supplemented with 0.4% glucose were diluted 1:1000 in M9 minimal medium and cultured anaerobically in an anaerobic hood using nitrogen and hydrogen as the background gas. The cultures were agitated for 24 hours, during which cells were grown by fumarate respiration using glucose (0.4%), fumarate (20 mM = 0.2%), or aspartate (2 mM) added to the medium as the electron acceptor. We estimated the growth capacity of each strain by measuring the maximum OD_600nm_ reached after 24 hours of culture under each condition.

### Determination of growth in urine

The human urine corresponds to a pool of urine obtained from three different healthy male volunteers who did not take any drugs or alcohol and were devoid of UTI history. An informative notice has been given to all volunteers according to the principles expressed in the declaration of Helsinki. The urine is double filtered using 0.45- and 0.2-μm filter units. Overnight cultures of *E. coli* 536 were diluted 1:1000 in refiltered urine supplemented or not with 2 mM aspartate. Cultures were then dispensed in 96-well plates. In each well, the volume of the culture was 150 μl of urine and 50 μl of mineral oil. During 20 hours of growth at 37°C with shaking, OD_600nm_ was measured every 5 minutes.

### UTI mouse model

We performed competition assays in the previously described mouse UTI model (*72*) between 536 and 536 Δ*pcm* strains. In competition assays, the relative ability of the two strains tested together to cause an ascending unobstructed UTI was determined from the bladder and kidney bacterial load. Two independent competition assays were performed using eight mice for each experiment. We used inducible fluorescent reporters to distinguish between 536 WT and Δ*pcm* strains. The two strains were inoculated at 1:1 final ratio. As a control, for each experiment, half of the mice were inoculated with a first mix composed of 536 *attB*_λ_::P*_lac_-mkate* and 536 Δ*pcm attB*_λ_::P*_lac_-gfp*, while the other mice were inoculated with a second mix composed of 536 *attB*_λ_::P*_lac_-gfp* and 536 Δ*pcm attB*_λ_::P*_lac_*-*mkate*. This experimental setup allowed us to verify that neither of the two fluorescent proteins influences CI in this assay. 536 Δ*pcm* relative ability to colonize the mouse urinary tract was determined 2 days after infection from the urine, the bladder, and the kidney bacterial load. Relative fitness of the mutant strain was estimated by calculating the CI: ${\mathrm{CI}}_{n}=\frac{{\text{Day}}_{n}\left(\%\text{mutant}/\text{WT}\right)}{{\text{Day}}_{0}\left(\%\text{mutant}/\text{WT}\right)}$.
