# Supplementary material for: The protein carboxymethyltransferase–dependent aspartate salvage pathway plays a crucial role in the intricate metabolic network of Escherichia coli
Source: Sci Adv. 2024 Feb 9;10(6):eadj0767. doi: 10.1126/sciadv.adj0767 (PMC10857468; doi:10.1126/sciadv.adj0767)
Supplement: Supplementary file 1 — Figs. S1 to S7 Tables S1 to S3 [file sciadv.adj0767_sm.pdf]

Science Advances
AAAS

Supplementary Materials for

The protein carboxymethyltransferase–dependent aspartate salvage pathway plays a crucial role in the intricate metabolic network of *Escherichia coli*

Maureen Micaletto *et al.*

Corresponding author: Ivan Matic, ivan.matic@inserm.fr

Sci. Adv. **10**, eadj0767 (2024)
DOI: 10.1126/sciadv.adj0767

This PDF file includes:

Figs. S1 to S7
Tables S1 to S3

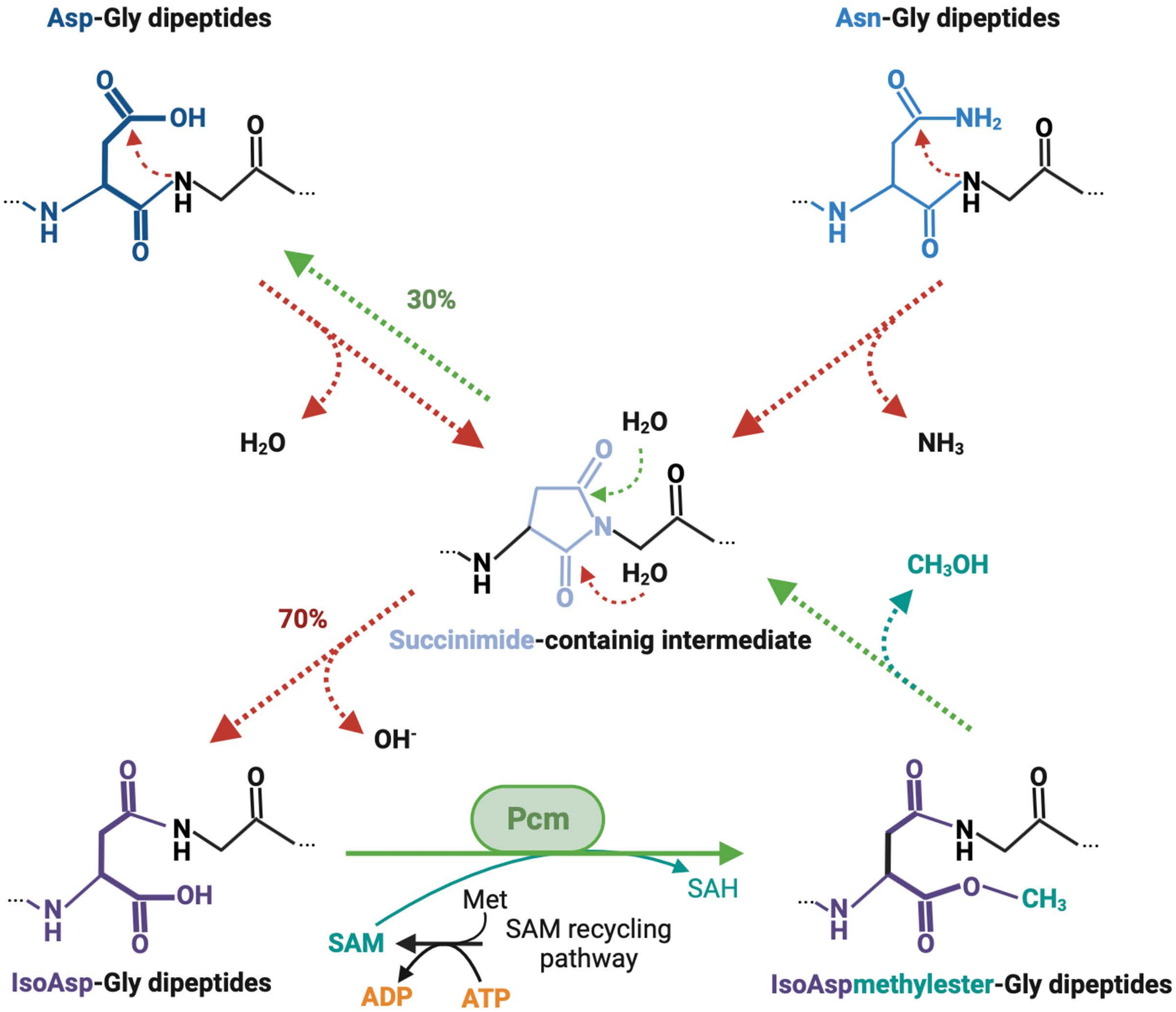
Asp-Gly dipeptides
Asn-Gly dipeptides
30%
H2O
NH3
H2O
H2O
Succinimide-containig intermediate
70%
CH3OH
OH-
Pcm
SAH
Met
SAM recycling pathway
SAM
ADP
ATP
IsoAsp-Gly dipeptides
IsoAspmethylester-Gly dipeptides

Fig. S1 | Formation and repair of isoaspartate mediated by Pcm enzyme.

When Aspartate (Asp) and asparagine (Asn) are found in peptides, such as aspartate-glycine dipeptides (Asp-Gly dipeptides) or in proteins, they are highly prone to non-enzymatic dehydration and deamidation processes, respectively. These processes lead to the formation of unstable succinimide intermediates, which subsequently undergo rapid and spontaneous hydrolysis, resulting in the formation of isoaspartate (IsoAsp). IsoAsp is processed by Pcm, which initiates its reparation by transferring a methyl group from a S-adenosyl-methionine (SAM) molecule to IsoAsp. The resulting L-IsoAspmethylester converts spontaneously back into succinimide. The spontaneous hydrolysis on one side of the ring nitrogen leads to Asp formation, while the cleavage on the other side of the ring nitrogen regenerate IsoAsp in a 3:1 ratio. Red arrows indicate the IsoAsp formation pathway, while green arrows indicate the IsoAsp repair pathway mediated by the Pcm enzyme. Dotted arrows correspond to spontaneous steps, and the green solid arrow corresponds to the Pcm-dependent step, which is the only enzymatic-driven step of the IsoAsp repair pathway. Methionine (Met), S-adenosylhomocysteine (SAM), S-adenosylhomocysteine (SAH).

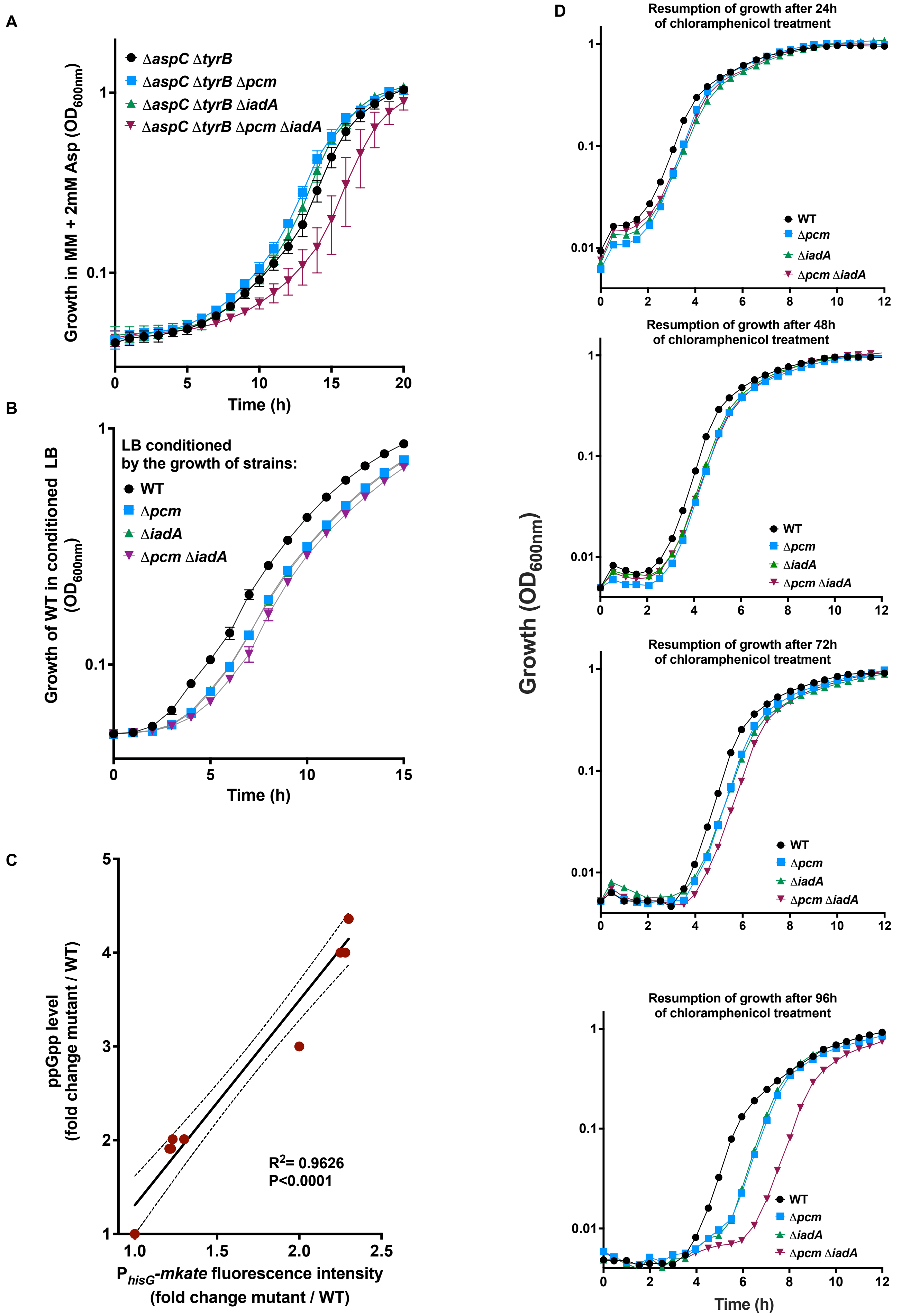

A
ΔaspC ΔtyrB
ΔaspC ΔtyrB Δpcm
ΔaspC ΔtyrB ΔiadA
ΔaspC ΔtyrB Δpcm ΔiadA
Growth in MM + 2mM Asp (OD600nm)
Time (h)
B
LB conditioned by the growth of strains:
WT
Δpcm
ΔiadA
Δpcm ΔiadA
Growth of WT in conditioned LB (OD600nm)
Time (h)
C
ppGpp level (fold change mutant / WT)
R2= 0.9626
P<0.0001
PhisG-mkate fluorescence intensity (fold change mutant / WT)
D
Resumption of growth after 24h of chloramphenicol treatment
Resumption of growth after 48h of chloramphenicol treatment
Resumption of growth after 72h of chloramphenicol treatment
Resumption of growth after 96h of chloramphenicol treatment
WT
Δpcm
ΔiadA
Δpcm ΔiadA
Growth (OD600nm)
Time (h)

Fig. S2 | Impact of Pcm on the maintenance of intracellular aspartate level, and on the capacity of cells to resume growth

(A) Growth of Δ*aspC* Δ*tyrB and its* Δ*pcm,* Δ*iadA* and Δ*pcm* Δ*iadA* derivatives strains, which are auxotroph for aspartate, in minimum medium M9 supplemented with 0.4% glucose and 2 mM of aspartate (MM+2 mM Asp). **(B)** Growth of WT strain in conditioned LB by the growth of WT, Δ*pcm,* Δ*iadA* or Δ*pcm* Δ*iadA.* **(C)** Correlation between the fold increase of the fluorescence intensity of the stringent response reporter P_{hisG}-*mkate* and the fold increase of ppGpp level from LC-MS measurement of mutant *vs*. WT strains at different time point during growth. **(D)** Resumption of growth after the antibiotic removal of WT, Δ*pcm,* Δ*iadA* and Δ*pcm* Δ*iadA* strains that have been treated with bacteriostatic antibiotic chloramphenicol (10XMIC) during 24h, 48h, 72h and 96h.

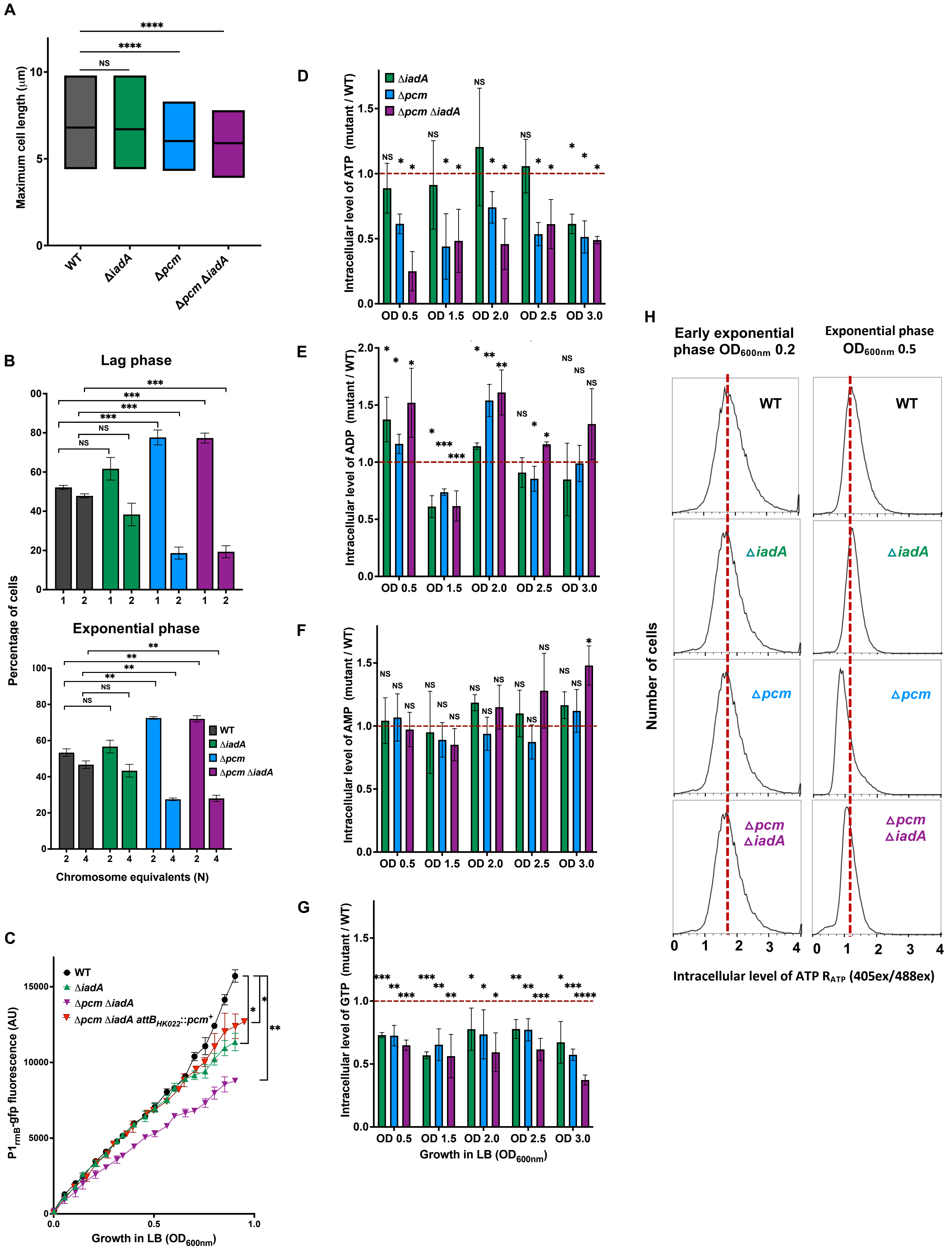
A
Maximum cell length (μm)
WT
ΔiadA
Δpcm
Δpcm ΔiadA
NS
****
****
B
Lag phase
Exponential phase
Percentage of cells
Chromosome equivalents (N)
WT
ΔiadA
Δpcm
Δpcm ΔiadA
C
WT
ΔiadA
Δpcm ΔiadA
Δpcm ΔiadA attB$_{HK022}$::pcm^{+}
P1$_{rrnB}$-gfp fluorescence (AU)
Growth in LB (OD$_{600nm}$)
D
ΔiadA
Δpcm
Δpcm ΔiadA
Intracellular level of ATP (mutant / WT)
OD 0.5
OD 1.5
OD 2.0
OD 2.5
OD 3.0
E
Intracellular level of ADP (mutant / WT)
F
Intracellular level of AMP (mutant / WT)
G
Intracellular level of GTP (mutant / WT)
Growth in LB (OD$_{600nm}$)
H
Early exponential phase OD$_{600nm}$ 0.2
Exponential phase OD$_{600nm}$ 0.5
WT
ΔiadA
Δpcm
Δpcm ΔiadA
Number of cells
Intracellular level of ATP R$_{ATP}$ (405ex/488ex)

Fig. S3 | Impact of Pcm on cell size, DNA replication, rRNA production and energy level **(A)** Maximum cell size during growth calculated from the "mother machine" microfluidics experiments for more than 250 individual cells. Boxes represent the minimum and the maximum, the midline indicates the median. Asterisks show significant difference compared to the WT. (Mann-Whitney test *P<0.05, **P<0.01, ***P<0.001, ****P<0.0001). **(B)** Lag phase or exponentially growing WT, Δ*pcm*, Δ*iadA* and Δ*pcm* Δ*iadA* cells were treated simultaneously with rifampicin and cephalexin. DNA content was measured after 90 min of incubation with both antibiotics. Number of genome equivalents (N) was calculated using stationary phase, 1N cells, as a reference. For lag phase, bar graphs represent the percentage of cells having one or two chromosome equivalents (1N or 2N). For exponential phase, bar graphs represent the percentage of cells having two or four chromosome equivalents (2N or 4N). Each bar graph represents the mean value (± SD) from three independent experiments. Asterisks show significant differences compared to the WT (t-test: *P < 0.05, **P < 0.01, ***P<0.001). **(C)** Expression of the unstable *gfp*-based transcriptional reporter $P1_{rrnB}$-*gfp*-asv, during exponential growth of WT, Δ*pcm* and Δ*pcm attBHK022*::*pcm*$^{+}$ strains in LB medium. Each data point represents the mean value (± SD) from three independent experiments. Asterisks show significant difference compared to the WT. (t-test: *P < 0.05, **P < 0.01). **d-g**, Relative intracellular level of ATP **(D),** ADP **(E)**, AMP **(F)** and GTP **(G)** in Δ*pcm* and/or Δ*iadA* strains *vs.* WT strain measured by LC-MS at different time points during growth in LB medium. The results are expressed as fold change between each mutant and WT cells at each time point. **(D-G)** Each bar graph represents the mean value (± SD) from three independent experiments. Asterisks show significant differences compared to a fold change of 1 for each time point (t-test: *P < 0.05, **P < 0.01, ***P<0.001). **(H)** Distribution of single-cell intracellular ATP level in living *E. coli* in WT, Δ*pcm,* Δ*iadA* and Δ*pcm* Δ*iadA* cells using QUEEN-2M biosensor[35]. ATP level was estimated at OD_{600nm} 0.2, considered as early exponential phase, and OD_{600nm} 0.5, considered as middle exponential phase. Each distribution corresponds to 60,000 cells or three independent experiments. We calculated the median for each distribution. At OD_{600nm} 0.2 the RATP (405ex/488ex) median for WT cells is 1.94, for Δ*pcm* cells the median is 1.75*,* Δ*iadA* cells the median is 1.9 and for Δ*pcm* Δ*iadA* cells the median is 1.78. We then performed Mann-Whitney test and showed that the ATP level distribution was significantly different for Δ*pcm* and Δ*pcm* Δ*iadA* and not for Δ*iadA* cells compared to WT cells. At OD_{600nm} 0.5 the RATP (405ex/488ex) median for WT cells is 1.23, for Δ*pcm* cells the median is 0.97*,* for Δ*iadA* cells the median is 1.20 and for Δ*pcm* Δ*iadA* cells the median is 0.99. We then performed Mann-Whitney test and showed that the ATP level distribution was significantly different for Δ*pcm* and Δ*pcm* Δ*iadA* and not for Δ*iadA* cells compared to WT cells. (Mann-Whitney test ***P<0.001).

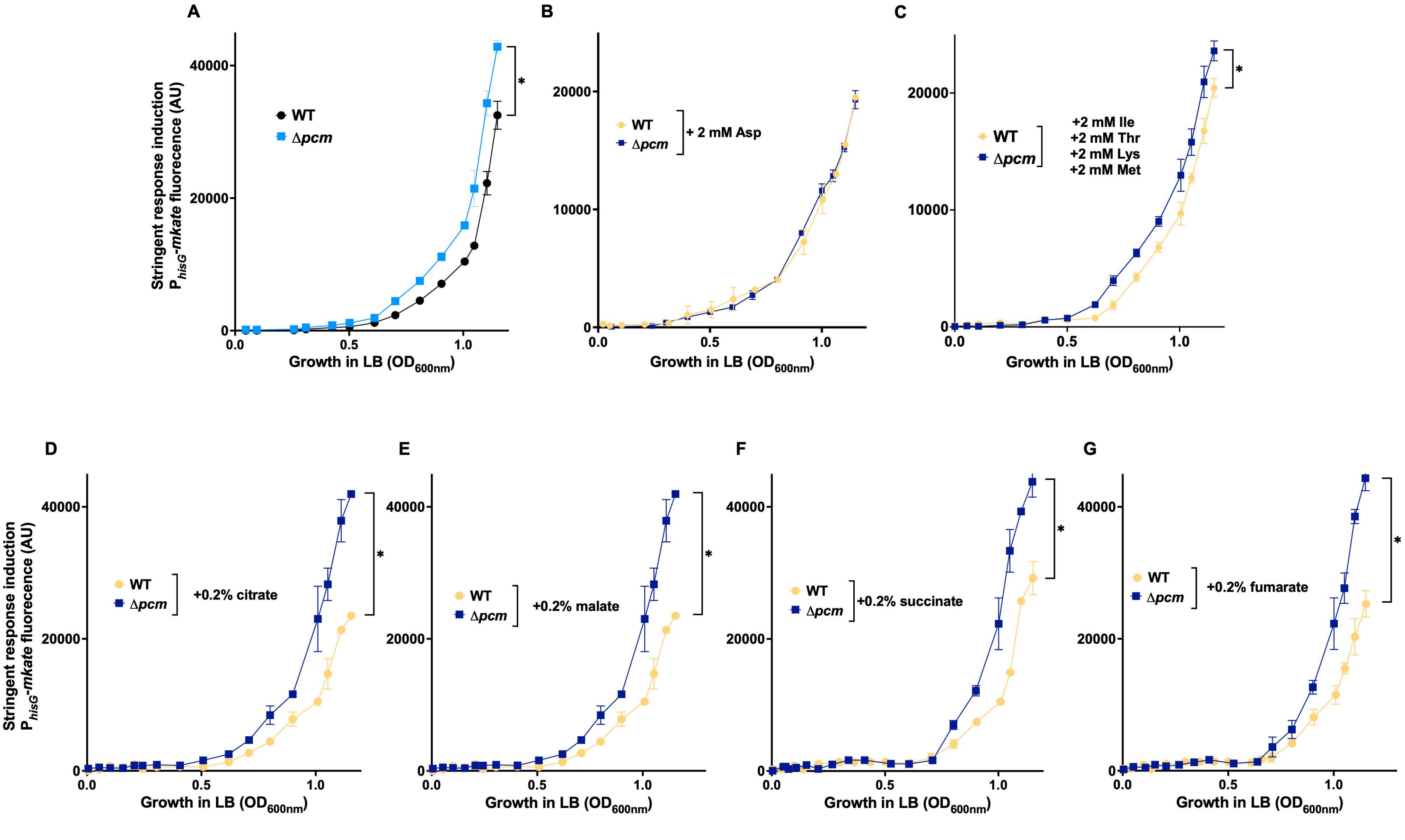

A
B
C
D
E
F
G
Stringent response induction
P_hisG-mkate fluorecence (AU)
Growth in LB (OD600nm)
WT
Δpcm
+ 2 mM Asp
+2 mM Ile
+2 mM Thr
+2 mM Lys
+2 mM Met
+0.2% citrate
+0.2% malate
+0.2% succinate
+0.2% fumarate
*

Fig. S4│ Effect of supplementation with Asp, various TCA cycle intermediates, or different amino acids on the stringent response induction.

(A-G) Expression of the stringent response induction reporter P_{hisG}-*mkate* during growth of WT and Δ*pcm* strains in LB medium supplemented or not **(A),** with 2 mM of aspartate (Asp) **(B),** 2 mM of isoleucine (Ile), threonine (Thr), lysine (Lys) and methionine (Met) **(C),** 0.2 % succinate **(D),** 0.2 % citrate **(E),** 0.2 % malate **(F),** or 0.2% fumarate **(G). (A-G),** Each data point represents the mean value (± SD) from three independent experiments. Asterisks show significant difference compared to the WT according to t-test (*P < 0.05, **P < 0.01).

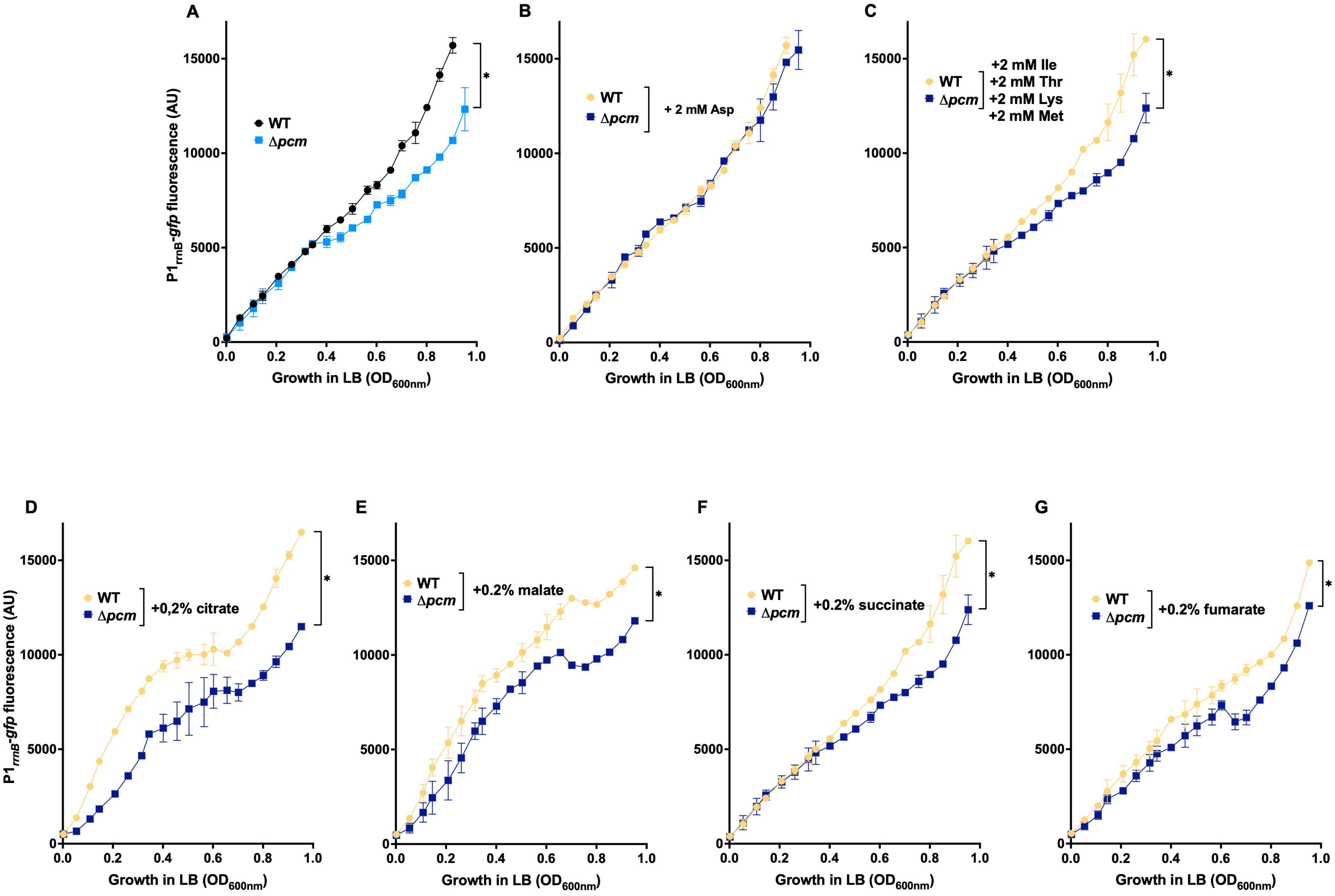
A
B
C
D
E
F
G
P1rrnB-gfp fluorescence (AU)
Growth in LB (OD600nm)
WT
Δpcm
+ 2 mM Asp
+2 mM Ile
+2 mM Thr
+2 mM Lys
+2 mM Met
+0,2% citrate
+0.2% malate
+0.2% succinate
+0.2% fumarate
*

Fig. S5| Effect of supplementation with Asp, various TCA cycle intermediates, or different amino acids on ribosomal RNA (rRNA) expression

(A-F) Expression of the transcriptional reporter P1$_{rrnB}$-*gfp*-asv, during exponential growth of WT, and Δ*pcm* strains in LB medium supplemented or not **(A),** with 2 mM of aspartate (Asp) **(B),** 2 mM of isoleucine (Ile), threonine (Thr), lysine (Lys) and methionine (Met) **(C),** 0.2 % succinate **(D),** 0.2 % citrate **(E),** 0.2 % malate **(F),** or 0.2% fumarate **(G). (A-G),** Each data point represents the mean value (± SD) from three independent experiments. Asterisks show significant difference compared to the WT according to t-test (*$P < 0.05$, **$P < 0.01$).

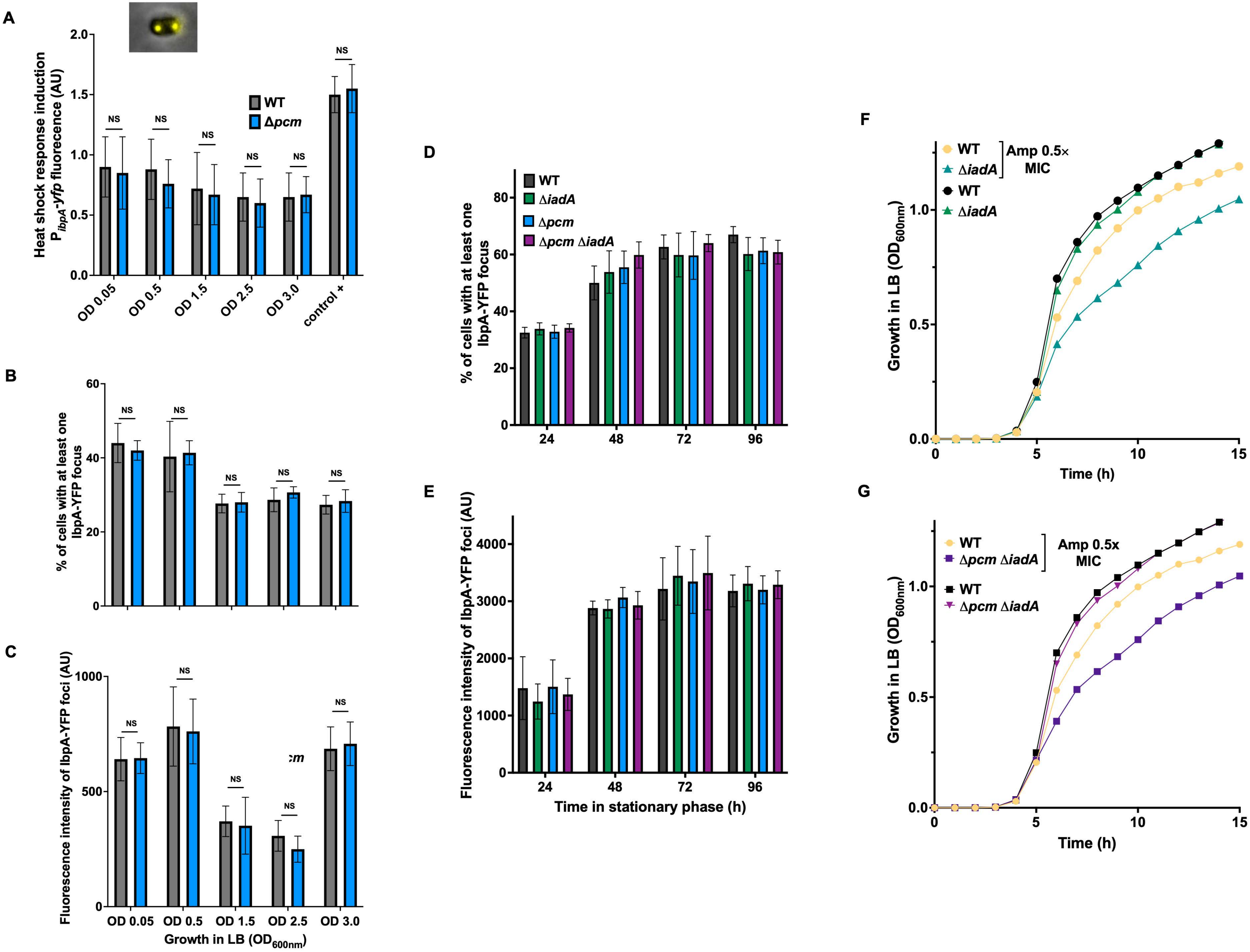
A
Heat shock response induction
P_{ibpA}-*yfp* fluorecence (AU)
WT
Δ*pcm*
NS
0.0
0.5
1.0
1.5
2.0
OD 0.05
OD 0.5
OD 1.5
OD 2.5
OD 3.0
control +
B
% of cells with at least one
IbpA-YFP focus
0
20
40
60
C
Fluorescence intensity of IbpA-YFP foci (AU)
0
500
1000
OD 0.05
OD 0.5
OD 1.5
OD 2.5
OD 3.0
Growth in LB (OD_{600nm})
D
% of cells with at least one
IbpA-YFP focus
WT
Δ*iadA*
Δ*pcm*
Δ*pcm* Δ*iadA*
0
20
40
60
80
24
48
72
96
E
Fluorescence intensity of IbpA-YFP foci (AU)
0
1000
2000
3000
4000
24
48
72
96
Time in stationary phase (h)
F
Growth in LB (OD_{600nm})
WT
Δ*iadA*
Amp 0.5× MIC
WT
Δ*iadA*
0.0
0.5
1.0
0
5
10
15
Time (h)
G
Growth in LB (OD_{600nm})
WT
Δ*pcm* Δ*iadA*
Amp 0.5x MIC
WT
Δ*pcm* Δ*iadA*
0.0
0.5
1.0
0
5
10
15
Time (h)

Fig. S6| Impact of Pcm on the level of heat shock response induction and protein aggregation during growth, and the growth of cells treated with sublethal doses of ampicillin.

(A) Induction of the heat shock response in WT and Δ*pcm* cells during growth. Heat shock response induction was quantified using a transcriptional reporter, P_{ibpA}-*yfp.* The *ibpA* gene codes for the Inclusion Body Protein A (IbpA), a small molecular chaperone, which is strongly expressed when the heat shock response is induced. Single cell fluorescence level associated with P_{ibpA} reporter was measured using flow cytometry. As a positive control, we examined the expression of the P_{ibpA}-*yfp* reporter after subjecting the cells to a 30-min exposure at 42°C, a treatment known to induce the heat shock response. Bar graphs represent the mean value (± SD) of fluorescence intensity calculated from three independent experiments. **b, c,** Detection of the protein aggregates. Cells carrying a IbpA-YFP translational reporter fusion were analyzed using a fluorescence microscope at different growth phases. **(B)** Bar graphs represent the percentage of WT and Δ*pcm* cells having at least one IbpA-YFP focus. **(C)** Bar graphs represent fluorescence intensity associated with each focus. **(B, C)** For each condition 1000 cells have been analyzed. (t-test: NS P>0.05). Inserted photograph shows IbpA-YFP foci in Δ*pcm* cells. **(D, E)** Detection of inclusion body in stationary phase cells carrying a IbpA-YFP reporter fusion were analyzed using a fluorescence microscope. **(D)** Bar graphs represent the percentage of WT, Δ*pcm,* Δ*iadA* and Δ*pcm* Δ*iadA* cells having at least one IbpA-YFP focus at different time of stationary phase. While we detected no significant difference between the different strains the proportion of cells with IbpA-YFP foci increased significantly over time compared to the percentage detected after 24h of stationary phase for all strains. **(E)** Bar graphs represent fluorescence intensity associated with each focus. While, we detected no significant difference between the different strains the fluorescence intensity associated with individual IbpA-YFP focus increased significantly over time compared to the fluorescence intensity after 24h of stationary phase for all strains. Total of 1,543, 1,432, 1,690 and 1,546 WT, Δ*pcm,* Δ*iadA* and Δ*pcm* Δ*iadA* cells respectively were analyzed (t-test: *P < 0.05, **P < 0.01, ***P<0.001). **(F, G)** Growth of WT, Δ*iadA* **(F)** and Δ*pcm* Δ*iadA* **(G)** in LB medium with or without 0.5 Minimum Inhibitory Concentration (MIC) of Ampicillin.

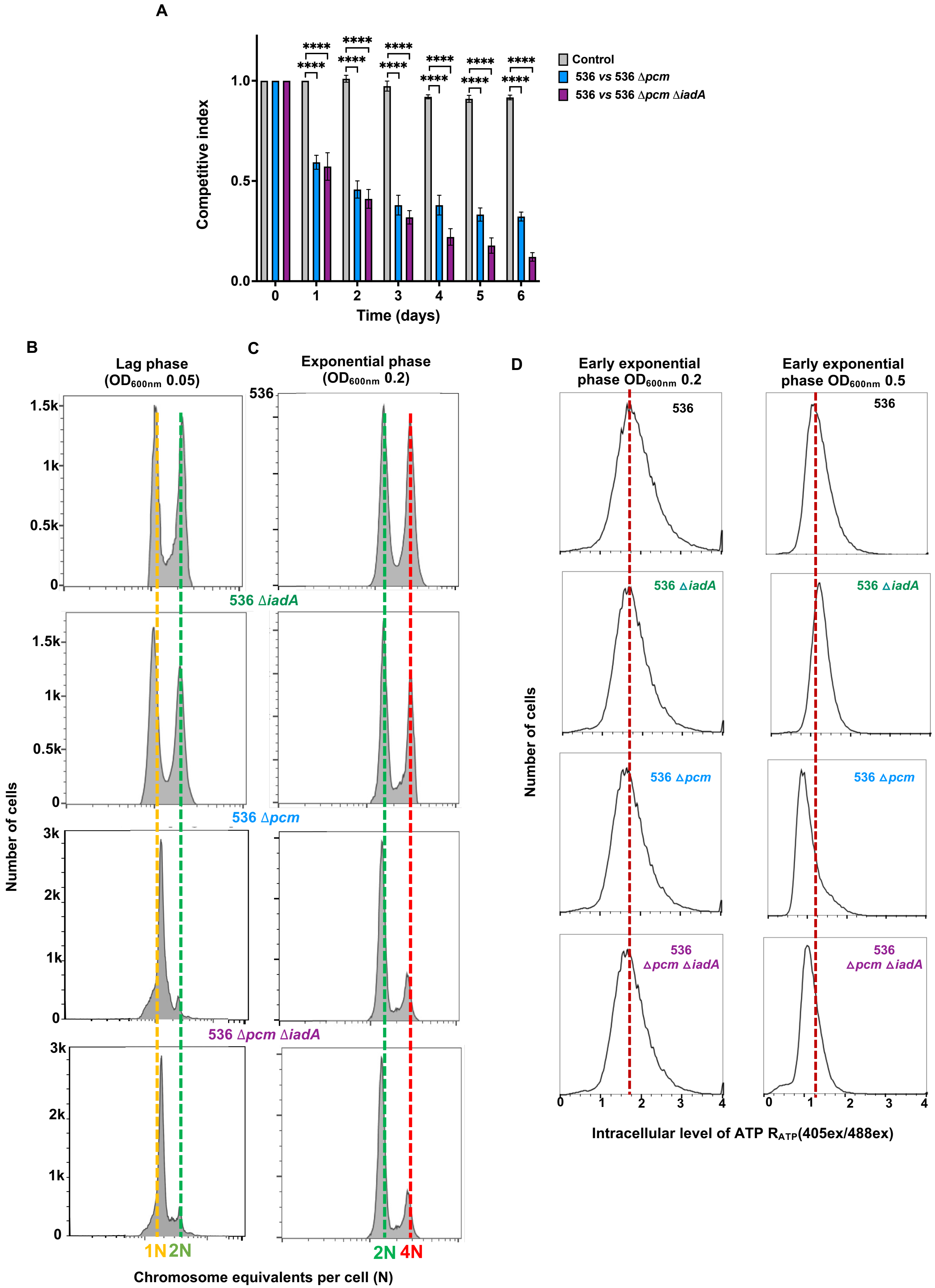

A
Competitive index
Time (days)
Control
536 vs 536 Δpcm
536 vs 536 Δpcm ΔiadA
B
Lag phase
(OD600nm 0.05)
C
Exponential phase
(OD600nm 0.2)
536
536 ΔiadA
536 Δpcm
536 Δpcm ΔiadA
1N 2N
2N 4N
Number of cells
Chromosome equivalents per cell (N)
D
Early exponential phase OD600nm 0.2
Early exponential phase OD600nm 0.5
536
536 ΔiadA
536 Δpcm
536 Δpcm ΔiadA
Number of cells
Intracellular level of ATP RATP(405ex/488ex)

Fig. S7 | Impact of Pcm on fitness, DNA replication and ATP level in *E. coli* 536

(A) Pairwise competitions Fitness of 536 *vs.* 536 Δ*pcm* or 536 Δ*pcm* Δ*iadA* cells measured by Fitness differences were estimated by calculating the competitive index. To verify that neither of the two fluorescent reporters influence the competitive ability of the strain, a control competition assay 536 Δ*pcm gfp vs*. 536 Δ*pcm mkate* was also performed. Bar graphs represent the mean value (± SD) of three independent experiments of competitive index analysis. Asterisks show significant differences compared to the control condition for each day according to t-test (*P < 0.05, **P < 0.01, ***P<0.001, ****P<0.0001). **(B, C)** Lag phase (first column) **(B)** or exponentially growing (second column) **(C)** 536, 536 Δ*pcm*, 536 Δ*iadA* and 536 Δ*pcm* Δ*iadA* cells were treated simultaneously with rifampicin and cephalexin. DNA content was measured after 90 min of incubation with both antibiotics. Number of genome equivalents (N) was calculated using stationary phase, 1N cells, as a reference. The results are representative of three independent experiments. **(D)** Distribution of single-cell intracellular ATP level in living *E. coli* in 536, 536 Δ*pcm*, 536 Δ*iadA* and 536 Δ*pcm* Δ*iadA* cells at OD_{600nm} 0.2 (considered as early exponential phase) and OD_{600nm} 0.5 (considered as middle exponential phase). Each distribution corresponds to 60,000 cells or three independent experiments. We calculated the median for each distribution. At OD_{600nm} 0.2 the RATP (405ex/488ex) median for WT cells is 1.84, for Δ*pcm* cells the median is 1.70*,* Δ*iadA* cells the median is 1.80 and for Δ*pcm* Δ*iadA* cells the median is 1.70. We then performed Mann-Whitney test and showed that the ATP level distribution was significantly different for Δ*pcm* and Δ*pcm* Δ*iadA* and not for Δ*iadA* cells compared to WT cells. At OD_{600nm} 0.5 the RATP (405ex/488ex) median for WT cells is 1.33, for Δ*pcm* cells the median is 1*,* Δ*iadA* cells the median is 1.20 and for Δ*pcm* Δ*iadA* cells the median is 1. We then performed Mann-Whitney test and showed that the ATP level distribution was significantly different for Δ*pcm* and Δ*pcm* Δ*iadA* and not for Δ*iadA* cells compared to WT cells. (Mann-Whitney test ***P<0.001).

Table S1: Bacterial strains and plasmids used in this study

Strain	Genotype	Source
***E. coli* MG1655**		
WT	MG1655 parental strain: *rph*-1λ^{-}	Laboratory strain collection
Δ*pcm*	*rph*-1λ^{-} Δ*pcm*::FRT	MG1655 P1 (KEIO Δ*pcm*::kan)
Δ*iadA*	*rph*-1λ^{-} Δ*iadA*::FRT	MG1655 P1 (KEIO Δ*iadA*::kan)
Δ*pcm* Δ*iadA*	*rph*-1λ^{-} Δ*pcm*::FRT Δ*iadA*::FRT	MG1655 P1 (KEIO Δ*pcm*::kan) P1 (KEIO Δ*iadA*::kan)
Δ*pcm* $attB_{HK022}$::*pcm*	*rph*-1λ^{-} Δ*pcm*::FRT $attB_{HK022}$::P_{pcm}-*pcm*	This study
Δ*pcm* Δ*iadA* $attB_{HK022}$::*pcm*	*rph*-1λ^{-} Δ*pcm*::FRT Δ*iadA*::FRT $attB_{HK022}$::P_{pcm}-*pcm*	This study
Δ*aspC* Δ*tyrB*	*rph*-1λ^{-} Δ*aspC*::FRT Δ*tyrB*::FRT	MG1655 P1 (KEIO Δ*aspC*::kan) P1 (KEIO Δ*tyrB*::kan)
Δ*pcm* Δ*aspC* Δ*tyrB*	*rph*-1λ^{-} Δ*pcm*::FRT Δ*aspC*::FRT Δ*tyrB*::FRT	Δ*pcm* P1 (KEIO Δ*aspC*::kan) P1 (KEIO Δ*tyrB*::kan)
Δ*iadA* Δ*aspC* Δ*tyrB*	*rph*-1λ^{-} Δ*pcm*::FRT Δ*aspC*::FRT Δ*tyrB*::FRT	Δ*iadA* P1 (KEIO Δ*aspC*::kan) P1 (KEIO Δ*tyrB*::kan)
Δ*pcm* Δ*iadA* Δ*aspC* Δ*tyrB*	*rph*-1λ Δ*pcm*::FRT Δ*iadA*::FRT Δ*aspC*::FRT Δ*tyrB*::FRT	Δ*pcm* Δ*iadA* P1 (KEIO Δ*aspC*::kan) P1 (KEIO Δ*tyrB*::kan)
Δ*mtn*	*rph*-1λ Δ*mtn*::*kan*	MG1655 P1 (KEIO Δ*mtn*::*kan)*
Δ*pcm* Δ*mtn*	*rph*-1λ^{-} Δ*pcm*::FRT Δ*mtn*::*kan*	Δ*pcm* P1 (KEIO Δ*mtn*::*kan)*

Δ*iadA* Δ*mtn*	*rph*-1λ^- Δ*iadA*::FRT Δ*mtn*::*kan*	Δ*iadA* P1 (KEIO Δ*mtn*::*kan)*
Δ*pcm* Δ*iadA* Δ*mtn*	*rph*-1λ^- Δ*pcm*::FRT Δ*iadA*::FRT Δ*mtn*::*kan*	Δ*pcm* Δ*iadA* P1 (KEIO Δ*mtn*::*kan)*
WT *intC* :: P_{hisG}-*mkate*	*rph*-1λ *intC* :: P_{hisG}-*mkate*	MG1655 P1 (*intC* :: P_{hisG}-*mkate-kan)*
Δ*pcm intC* :: P_{hisG}-*mkate*	*rph*-1λ^- Δ*pcm*::FRT *intC* :: P_{hisG}-*mkate*	Δ*pcm* P1 (*intC* :: P_{hisG}-*mkate-kan)*
Δ*iadA intC* :: P_{hisG}-*mkate*	*rph*-1λ^- Δ*iadA*::FRT *intC* :: P_{hisG}-*mkate*	Δ*iadA* P1 (*intC* :: P_{hisG}-*mkate-kan)*
Δ*pcm* Δ*iadA intC* :: P_{hisG}-*mkate*	*rph*-1λ^- Δ*pcm*::FRT Δ*iadA*::FRT *intC* :: P_{hisG}-*mkate*	Δ*pcm* Δ*iadA* P1 (*intC* :: P_{hisG}-*mkate-kan)*
WT *intC* :: P_{hisG}-*mkate* Δ*relA*	*rph*-1λ *intC* :: P_{hisG}-*mkate* Δ*relA*::FRT	MG1655 P1 (KEIO Δ*relA*::*kan)* P1 (*intC* :: P_{hisG}-*mkate-kan)*
Δ*pcm intC* :: P_{hisG}-*mkate* Δ*relA*	*rph*-1λ^- Δ*pcm*::FRT *intC* :: P_{hisG}-*mkate* Δ*relA*::FRT	Δ*pcm* P1 (KEIO Δ*relA*::*kan)* P1 (*intC* :: P_{hisG}-*mkate-kan)*
Δ*iadA intC* :: P_{hisG}-*mkate* Δ*relA*	*rph*-1λ^- Δ*iadA*::FRT *intC* :: P_{hisG}-*mkate* Δ*relA*::FRT	Δ*iadA* P1 (KEIO Δ*relA*::*kan)* P1 (*intC* :: P_{hisG}-*mkate-kan)*
Δ*pcm* Δ*iadA intC* :: P_{hisG}-*mkate* Δ*relA*	*rph*-1λ^- Δ*pcm*::FRT Δ*iadA*::FRT *intC* :: P_{hisG}-*mkate* Δ*relA*::FRT	Δ*pcm* Δ*iadA* P1 (KEIO Δ*relA*::*kan)* P1 (*intC* :: P_{hisG}-*mkate-kan)*
Δ*relA* Δ*spoT intC* :: P_{hisG}-*mkate*	*rph*-1λ^- Δ*relA*::FRT Δ*spoT207*::cat *intC* :: P_{hisG}-*mkate*	This study
WT *intC* :: PR-*mkate*	*rph*-1λ *intC* :: PR-*mkate*	MG1655 P1 (*intC* :: PR-*mkate-cat)*
WT *intC* :: PR-*gfp*	*rph*-1λ *intC* :: PR-*gfp*	MG1655 P1 (*intC* :: PR-*gfp-cat)*
Δ*pcm intC* :: PR-*mkate*	*rph*-1λ^- Δ*pcm*::FRT *intC* :: PR-*mkate*	Δ*pcm* P1 (*intC* :: PR-*mkate-cat)*

Δ*pcm intC* :: PR-*gfp*	*rph*-1λ⁻ Δ*pcm*::FRT *intC* :: PR-*gfp*	Δ*pcm* P1 (*intC* :: PR-*gfp-cat)*
Δ*iadA intC* :: PR-*mkate*	*rph*-1λ⁻ Δ*iadA*::FRT *intC* :: PR-*mkate*	Δ*iadA* P1 (*intC* :: PR-*mkate-cat)*
Δ*iadA intC* :: PR-*gfp*	*rph*-1λ⁻ Δ*iadA*::FRT *intC* :: PR-*gfp*	Δ*iadA* P1 (*intC* :: PR-*gfp-cat)*
Δ*pcm* Δ*iadA intC* :: PR-*mkate*	*rph*-1λ⁻ Δ*pcm*::FRT Δ*iadA*::FRT *intC* :: PR-*mkate*	Δ*pcm* Δ*iadA* P1 (*intC* :: PR-*mkate-cat)*
Δ*pcm* Δ*iadA intC* :: PR-*gfp*	*rph*-1λ⁻ Δ*pcm*::FRT Δ*iadA*::FRT *intC* :: PR-*gfp*	Δ*pcm* Δ*iadA* P1 (*intC* :: PR-*gfp-cat)*
WT $attB_{\lambda}$ :: $P1_{rrnB}$-*gfp*	*rph*-1λ $attB_{\lambda}$ :: $P1_{rrnB}$-*gfp*-ASV	MG1655 P1 ($attB_{\lambda}$ :: $P1_{rrnB}$-*gfp*-ASV-*kan)*
Δ*pcm* $attB_{\lambda}$ :: $P1_{rrnB}$-*gfp*	*rph*-1λ⁻ Δ*pcm*::FRT $attB_{\lambda}$ :: $P1_{rrnB}$-*gfp*-ASV	Δ*pcm* P1 ($attB_{\lambda}$ :: $P1_{rrnB}$-*gfp*-ASV-*kan)*
Δ*iadA* $attB_{\lambda}$ :: $P1_{rrnB}$-*gfp*	*rph*-1λ⁻ Δ*iadA*::FRT $attB_{\lambda}$ :: $P1_{rrnB}$-*gfp*-ASV	Δ*iadA* P1 ($attB_{\lambda}$ :: $P1_{rrnB}$-*gfp* -ASV-*kan)*
Δ*pcm* Δ*iadA* $attB_{\lambda}$ :: $P1_{rrnB}$-*gfp*	*rph*-1λ⁻ Δ*pcm*::FRT Δ*iadA*::FRT $attB_{\lambda}$ :: $P1_{rrnB}$-*gfp*-ASV	Δ*pcm* Δ*iadA* P1 ($attB_{\lambda}$ :: $P1_{rrnB}$-*gfp*-ASV-*kan)*
Δ*pcm* $attB_{\lambda}$ :: $P1_{rrnB}$-*gfp* $attB_{HK022}$::*pcm*	*rph*-1λ⁻ Δ*pcm*::FRT $attB_{\lambda}$ :: $P1_{rrnB}$-*gfp*-ASV $attB_{HK022}$::P_{pcm}-*pcm*	Δ*pcm* $attB_{\lambda}$ :: $P1_{rrnB}$-*gfp* ASV-*kan* P1 ($attB_{HK022}$::P_{pcm}-*pcm-cat)*
Δ*pcm* Δ*iadA* $attB_{\lambda}$ :: $P1_{rrnB}$-*gfp* $attB_{HK022}$::*pcm*	*rph*-1λ⁻ Δ*pcm*::FRT Δ*iadA*::FRT $attB_{\lambda}$ :: $P1_{rrnB}$-*gfp*-ASV $attB_{HK022}$::P_{pcm}-*pcm*	Δ*pcm* Δ*iadA* $attB_{\lambda}$ :: $P1_{rrnB}$-*gfp*-ASV-*kan* P1 ($attB_{HK022}$::P_{pcm}-*pcm-cat)*
WT P_{ibpA}-*yfp*	*rph*-1λ⁻ *intC* :: P_{ibpA}-*yfp*	This study
Δ*pcm* P_{ibpA}-*yfp*	*rph*-1λ⁻ Δ*pcm*::FRT *intC* :: P_{ibpA}-*yfp*	This study
WT IbpA-YFP	*rph*-1λ⁻ *ibpA*::*ibpA-yfp*	(39)
Δ*pcm* IbpA-YFP	*rph*-1λ⁻ Δ*pcm*::FRT *ibpA*::*ibpA-yfp*	This study

Δ*iadA* IbpA-YFP	*rph*-1λ⁻ Δ*iadA*::FRT *ibpA*::*ibpA-yfp*	This study
Δ*pcm* Δ*iadA* IbpA-YFP	*rph*-1λ⁻ Δ*pcm*::FRT Δ*iadA*::FRT *ibpA*::*ibpA-yfp*	This study
WT Queen-2m	*rph*-1λ⁻ + pWL2-QUEEN-2m	This study
Δ*pcm* Queen-2m	*rph*-1λ⁻ Δ*pcm*::FRT + pWL2-QUEEN-2m	This study
Δ*iadA* Queen-2m	*rph*-1λ⁻ Δ*iadA*::FRT + pWL2-QUEEN-2m	This study
Δ*pcm* Δ*iadA* Queen-2m	*rph*-1λ⁻ Δ*pcm*::FRT Δ*iadA*::FRT + pWL2-QUEEN-2m	This study
	***E. coli* 536**	
WT_{536}	536 parental strain	Laboratory strain collection
Δpcm_{536}	Δ*pcm*::FRT	This study
Δ$iadA_{536}$	Δ*iadA*::FRT	This study
Δ*pcm* Δ$iadA_{536}$	Δ*pcm*::FRT Δ*iadA*::FRT	This study
WT_{536}	536 parental strain + pWL2-QUEEN-2m	This study
Δpcm_{536}	Δ*pcm*::FRT + pWL2-QUEEN-2m	This study
Δ$iadA_{536}$	Δ*iadA*::FRT + pWL2-QUEEN-2m	This study
Δ*pcm* Δ$iadA_{536}$	Δ*pcm*::FRT Δ*iadA*::FRT + pWL2-QUEEN-2m	This study
WT_{536} $attB_{\lambda}$ :: PL_{lacO1}-*gfp*	$attB_{\lambda}$ :: PL_{lacO1}-*gfp-cat*	This study
WT_{536} $attB_{\lambda}$ :: PL_{lacO1}-*mkate*	$attB_{\lambda}$ :: PL_{lacO1}-*mkate-cat*	This study
Δpcm_{536} $attB_{\lambda}$ :: PL_{lacO1}-*gfp*	Δ*pcm*::FRT $attB_{\lambda}$ :: PL_{lacO1}-*gfp-cat*	This study
Δpcm_{536} $attB_{\lambda}$ :: PL_{lacO1}-*mkate*	Δ*pcm*::FRT $attB_{\lambda}$ :: PL_{lacO1}-*mkate-cat*	This study

Δ*pcm* Δ*iadA*$_{536}$ *attB*$_{\lambda}$ :: P*L*$_{lacO1}$-*gfp*	Δ*pcm*::FRT Δ*iadA*::FRT *attB*$_{\lambda}$ :: P*L*$_{lacO1}$-*gfp-cat*	This study
Δ*pcm* Δ*iadA*$_{536}$ *attB*$_{\lambda}$ :: P*L*$_{lacO1}$-*mkate*	Δ*pcm*::FRT Δ*iadA*::FRT *attB*$_{\lambda}$ :: P*L*$_{lacO1}$-*mkate-cat*	This study
Plasmids		
pKD46		(66)
pKD3		(66)
pCP20		(66)
pLDR8		This study
pLDR10		This study
pOSIP-CH		(67)
pWL2-QUEEN-2m		(35)

Table S2: Primers used in this study

Name	Primer sequence (5'-3')
P$_{pcm}$_complementation-up	CCGGTACCATTGTTGCTGAGCGCCTCAA
P$_{pcm}$_complementation-dw	CCACTAGTAATCACCTGTTGTGTTGATA
*pcm*_complementation-up	CCACTAGTatgGTAAGCAGACGCGTACA
*pcm*_complementation-dw	CCGCATGCAGCCAGCTCACCCTTCA
536_*pcm*_deletion-up	AGCAGACGCGTACAAGCACTTCTGGATCA ATTACGTGCGC
536_*pcm*_deletion-dw	ttaAGCCAGCTCACCCTTCACTAAAGGGAC AAAGCGCACG
536_*iadA*_deletion-up	atgATTGATTATTCCGCAGCCGGTTTTACCC TGCTGCAGG
536_*iadA*_deletion-dw	ttaTGCCGTTTCA AACGTTCCTTTCACGCAG GCTTTGCCG CATATGAATATCCTCCTTAG

<u>Supplementary table 3</u> Growth parameters of cells monitored using microfluidic "mother machine" device.

	**WT**			**Δ*pcm***				**Δ*iadA***				**Δ*pcm* Δ*iadA***			
Number of cells	287			266				260				267			
	**Median**	**Mean ± SD**	**CV (%)**	**Median**	**Mean ± SD**	**CV (%)**	**Ratio relative to WT**	**Median**	**Mean ± SD**	**CV (%)**	**Ratio relative to WT**	**Median**	**Mean ± SD**	**CV (%)**	**Ratio relative to WT**
**Lag time (min)**	**50**	**53.71 ± 6.64**	**12.36**	**65**	**65.21 ± 7.589**	**11.64**	**1.3**	**60**	**59,23 ± 6.64**	**11.21**	**1.2**	**70**	**67.33 ± 8.615**	**12.74**	**1.4**
**Division time (min)**	**15**	**17.14 ± 4.074**	**23.77**	**20**	**21.33 ± 5.898**	**27.07**	**1.25**	**20**	**18.3 ± 6.001**	**23.08**	**1.1**	**20**	**22.33 ± 3.595**	**26.88**	**1.3**
**Cell size before division (µm)**	**6.7**	**6.69 ± 0.9655**	**15.98**	**6**	**6.246 ± 1.013**	**15.68**	**0.89**	**6.8**	**6.73 ± 0.8315**	**15.18**	**1.01**	**5.9**	**5.944 ± 1.012**	**17.88**	**0.88**

	**Δ*pcm attB*$_{HK022}$::*pcm*$^{+}$**				**Δ*pcm* Δ*iadA attB*$_{HK022}$::*pcm*$^{+}$**			
Number of cells	255				251			
	**Median**	**Mean ± SD**	**CV (%)**	**Ratio relative to WT**	**Median**	**Mean ± SD**	**CV (%)**	**Ratio relative to WT**
**Lag time (min)**	**50**	**51.84 ± 6.318**	**14.12**	**1.05**	**55**	**55.68 ± 6.277**	**13.7**	**1.1**
**Division time (min)**	**15**	**17.81 ± 5.376**	**23.19**	**1.1**	**15**	**17.08 ± 5.246**	**23.74**	**0.99**
**Cell size before division (µm)**	**6.75**	**6.70 ± 0.8547**	**15.58**	**1.02**	**6.8**	**6.75 ± 0.7612**	**15.39**	**1.01**
